# Carbon Monoxide Controllable Targeted Gas Therapy for Synergistic Anti-inflammation

**DOI:** 10.1016/j.isci.2020.101483

**Published:** 2020-08-20

**Authors:** Chun Liu, Zhi Du, Mengmeng Ma, Yuhuan Sun, Jinsong Ren, Xiaogang Qu

**Affiliations:** 1 Laboratory of Chemical Biology and State Key Laboratory of Rare Earth Resource Utilization, Changchun Institute of Applied Chemistry, Chinese Academy of Sciences, Changchun, Jilin 130022, China; 2 University of Science and Technology of China, Hefei, Anhui 230026, China; 3 University of Chinese Academy of Sciences, Beijing 100039, China

**Keywords:** Chemistry, Biochemistry, Nanotechnology

## Abstract

Carbon monoxide (CO) plays an important role in the regulation of a variety of physiological processes and thus is regarded as a promising pharmaceutical agent. Nevertheless, therapeutic applications of CO are severely hampered by the difficulty of the delivery of controlled amounts of CO to biological targets. To address this deficiency, we present a spatiotemporally controllable CO-releasing platform (designated as Neu-MnO_2_/Fla) for synergistic anti-inflammation. With the assistance of neutrophil membrane coating, Neu-MnO_2_/Fla can target to inflammatory sites. Subsequently, excess H_2_O_2_ at the inflamed tissues can be decomposed into oxygen because of MnO_2_ as nanozymes possessing catalase (CAT) activity, which not only relieves oxidative stress but also achieves *in situ* rapid photo-induced CO release. The *in vitro* and *in vivo* results indicate our CO-releasing platform exhibits a strong synergistic anti-inflammatory effect. Our work will shed light on targeted CO release to avoid side effects of therapeutic applications of CO.

## Highlights

• The Neu-MnO_2_/Fla was successfully constructed
• Neu-MnO_2_/Fla can deliver CO to inflammatory sites in a safe and effective way
• Synergistic anti-inflammatory effects were achieved by combining MnO_2_ and CO
• The Neu-MnO_2_/Fla reduced the level of ROS and pro-inflammatory cytokines

## Graphical Abstract

**Figure:**
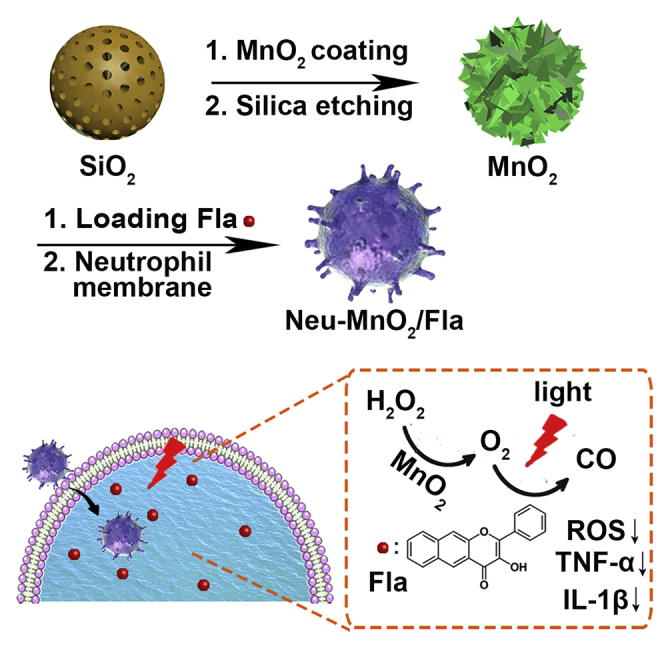

## Introduction

The emerging researches of carbon monoxide (CO) gas therapy have attracted widespread attention, including therapy of neurodegenerative diseases (Queiroga et al., 2015) and as an antibacterial (Wareham et al., 2015), anti-cancer (Wegiel et al., 2013; Wu et al., 2018a), and especially anti-inflammatory agent (He et al., 2015; Ji et al., 2016b; Popova et al., 2018; Zheng et al., 2018; Wang et al., 2019). Moreover, studies have revealed that CO serves as an endogenous signaling molecule and shows significant anti-inflammatory effects at low doses in many inflammation-related diseases (Otterbein et al., 2000). However, the clinical application of inhaled CO presents several disadvantages, including lack of tissue specificity, difficulty in controlling precise amounts, and the need for complex hospital devices (Ji et al., 2016a). To overcome these limitations, many CO-releasing molecules (CORMs) have been developed as non-gaseous forms of CO delivery (Mann, 2012; Heinemann et al., 2014; Garcia-Gallego and Bernardes, 2014; Chakraborty et al., 2014; Chaves-Ferreira et al., 2015). Yet, most of these CORMs are transition metal carbonyl complexes, which often show potential toxicity and background CO release (Kautz et al., 2016). Fortunately, emerging organic CORMs offer an alternative way for safer and more effective CO delivery (Popova et al., 2018; Zheng et al., 2018; Li et al., 2018b; Ji and Wang, 2018). Recently, Berreau's group reported a flavonol-based organic CORM, 3-hydroxybenzo [*g*]flavone (Fla), featuring the capacities of fluorescence traceability in cells, low toxicity, and CO release in the presence of oxygen and the light (Popova et al., 2018; Anderson et al., 2015; Soboleva et al., 2017).

However, these small molecules like CORMs usually exhibit unsatisfactory biological effects because of their random biodistribution, fast renal excretion, poor tissue permeability, and low retention at lesion areas (Ji et al., 2016a; Garcia-Gallego and Bernardes, 2014; Li et al., 2018a). To address the aforementioned challenges, various nanocarriers have been constructed for drug delivery (He et al., 2015; Hasegawa et al., 2010; Yao et al., 2018a; Yang et al., 2017). In particular, hollow manganese dioxide (MnO_2_) nanoparticles are promising drug carriers owing to high drug-loading capacity, intelligent biodegradability (Fan et al., 2016; Yang et al., 2018), and inherent catalase (CAT) mimic activity (Wan et al., 2012; Li et al., 2017). On the one hand, MnO_2_ nanocarriers can deliver these drugs safely and effectively, avoiding rapid pervasion within the body after administration; on the other hand, MnO_2_ as nanozymes possessing catalase (CAT) activity can efficiently decompose the endogenous H_2_O_2_ to evolve ample oxygen to boost *in situ* CO release in inflamed tissues. Moreover, MnO_2_ nanoparticles can be decomposed to biocompatible Mn^2+^ ions rapidly discharged by kidneys. Hence, there should be no long-term toxicity issues for MnO_2_ nanoparticles as a therapeutic agent in biological systems (Yang et al., 2017; Zhu et al., 2016; Chen et al., 2016). Taking these advantages into consideration, we aim to develop a CO delivery platform based on hollow mesoporous MnO_2_ nanozymes.

Neutrophils, a type of white blood cells, can autonomously move along the chemotactic gradients toward the inflammatory sites (behavior known as chemotaxis) (Xue et al., 2017; Shao et al., 2017; Wu et al., 2018b; Zhang et al., 2019). Thus, neutrophil cell membrane-derived nanoparticles have been reported as a promising targeted drug delivery platform for many autoimmune diseases and inflammatory disorders (Yurkin and Wang, 2017; Chu et al., 2018; Zhang et al., 2018).

**Figure 1:**
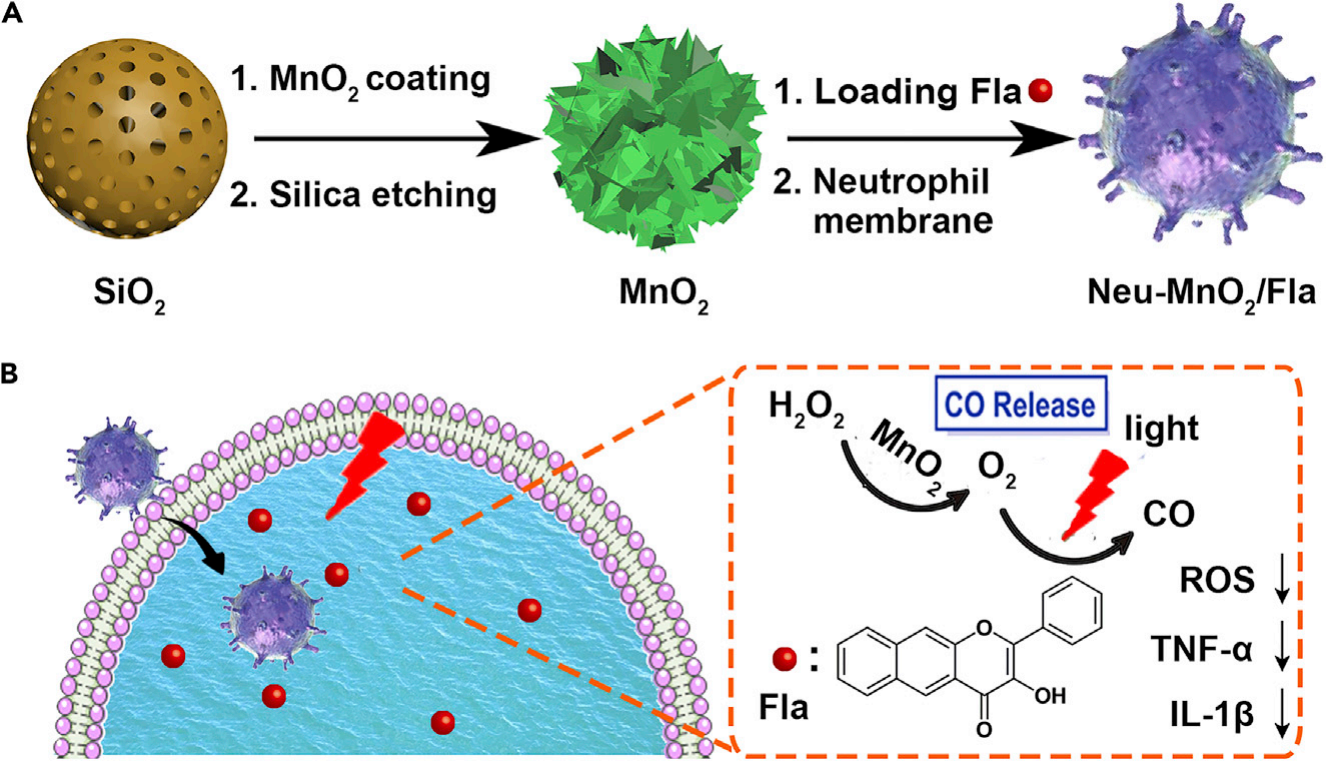
Schematic Representation of Synergistic Anti-inflammation Schematic illustration of (A) the preparation of Neu-MnO2/Fla and (B) neutrophil membrane-targeted *in situ* CO release for synergistic anti-inflammatory effects promoted by MnO2 nanozymes.

By the integration of Fla, MnO_2,_ and neutrophil membrane, we presented a CO-releasing nano-platform for *in vivo* synergistic anti-inflammation, which could achieve targeted CORMs delivery and spatiotemporally controllable *in situ* CO release. As illustrated in Figure 1, the hollow mesoporous MnO_2_ nanoparticles were chosen as the carriers and used for encapsulating Fla, a small molecule prodrug for CO release (Anderson et al., 2015). Then the MnO_2_ nanoparticles were further coated with neutrophil cell membrane to obtain Neu-MnO_2_/Fla. In a lipopolysaccharide (LPS)-induced inflammation model, the subsequently administrated Neu-MnO_2_/Fla were primed by the chemoattractants and migrated to the inflammatory sites. Afterward, Neu-MnO_2_/Fla achieved *in situ* rapid photo-induced CO release in the presence of ample oxygen and the light and thus produced a significant synergistic anti-inflammatory effect.

## Results and Discussion

**Figure 2:**
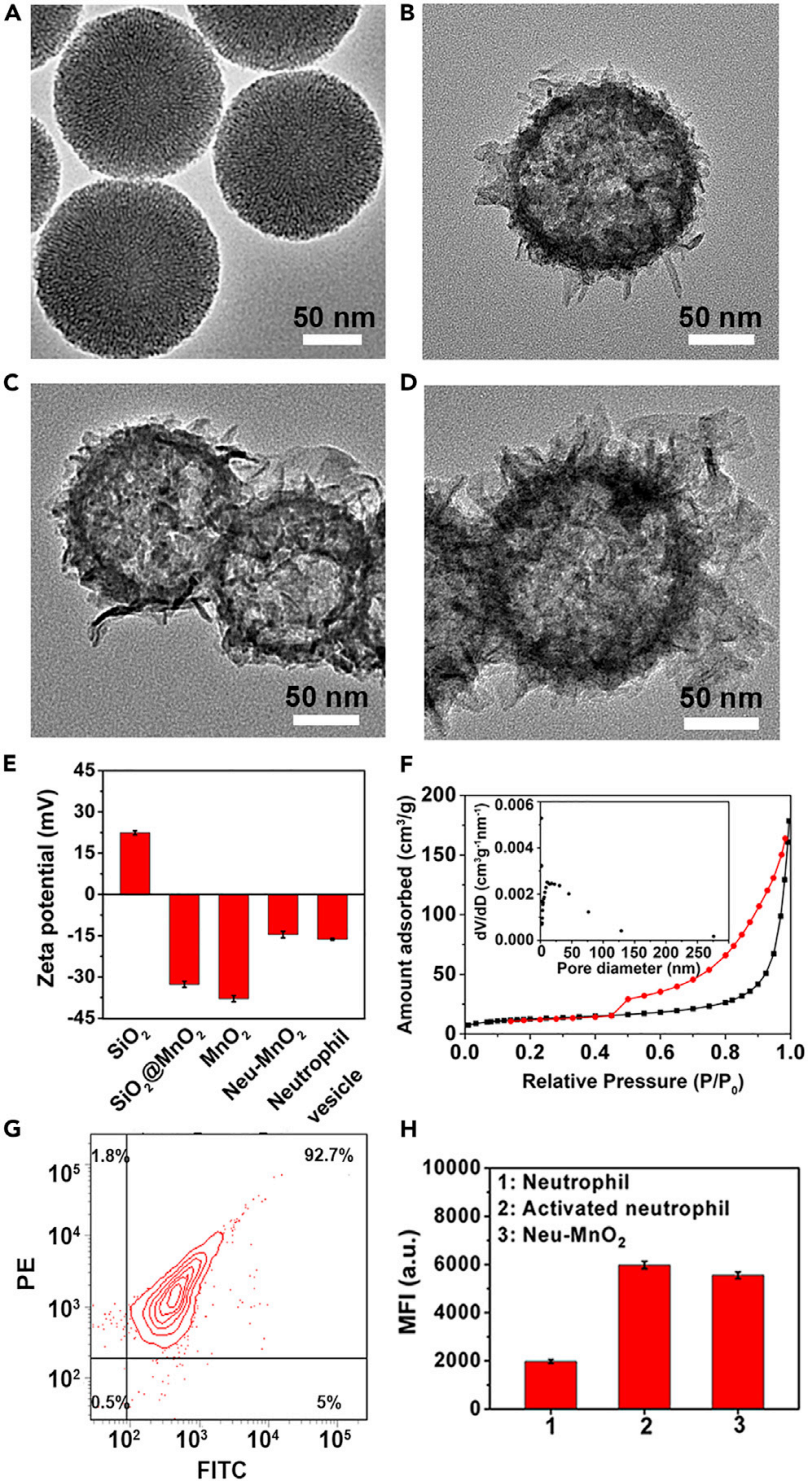
Synthesis and Characterization of Neu-MnO2/Fla (A–D) TEM images of (A) SiO2, (B) SiO2@MnO2, (C) MnO2, and (D) Neu-MnO2. The ethanol solution of the samples was dripped onto the Formvar Stabilized with Carbon Support Films for the test. (E) Zeta potential of SiO2, SiO2@MnO2, MnO2, Neu-MnO2, and Neutrophil vesicle. The samples were dispersed in an aqueous solution. (F) Nitrogen adsorption-desorption curves of the MnO2. Inset figure: pore size distributions derived from the adsorption branch according to the BJH model. (G) Flow cytometry analysis of the purity of neutrophils co-stained with anti-Ly6g antibody (FITC) and PE-anti rat CD11b/c antibody (OX-42). The lower-left, lower-right, upper-left, and upper-right quadrants represent the populations of FITC-/PE-, FITC+/PE-, FITC-/PE+, and FITC+/PE + cells, respectively. (H) Mean fluorescence intensity (MFI) measured in neutrophils, activated neutrophils, and Neu-MnO2 stained with PE-anti rat CD11b/c antibody (OX-42). The results are presented as means ± standard deviation from three independent experiments.

In order to verify our hypothesis, the Neu-MnO_2_/Fla were synthesized by embedding Fla into the hollow mesoporous MnO_2_ nanoparticles and further coating them with neutrophil membrane. The mesoporous silica nanoparticles (SiO_2_) with uniform spherical morphology were synthesized as the templates (Figures 2A and S1). Uniform mesoporous MnO_2_ layers were deposited on the surface of SiO_2_ (SiO_2_@MnO_2_) through a hydrothermal process (Figure 2B). After etching silica, uniform hollow mesoporous MnO_2_ nanoparticles were obtained (Figure 2C). Elemental mapping further confirmed the hollow structure of MnO_2_ nanoparticles (Figure S2). The surface zeta potential transformed from positive to negative after MnO_2_ coating and more negative after silica etching treatment (Figure 2E). Dynamic light scattering (DLS) demonstrated the hydrodynamic diameters of MnO_2_ increased after neutrophil membrane coating (Figure S3). Besides, the Fourier transform infrared (FTIR) spectrum and X-ray photoelectron spectroscopy (XPS) analysis additionally indicated the preparation of MnO_2_ (Figures S4 and S5). X-ray diffraction (XRD) patterns showed that the diffraction peaks could match well with the crystal phase of δ-MnO_2_ (JCPDS No. 80-1098, Figure S6). Nitrogen adsorption-desorption curves of the MnO_2_ were measured. Figure 2F showed a pronounced hysteresis at higher p/p_0_ as a result of the creation of mesoporosity. The created mesopores showed a broad distribution centered around approximately 10.80 nm. The hollow and mesoporous structures made them ideal for efficient drug loading. To employ the MnO_2_ nanoparticles for safe and effective CO delivery, a photo-induced CORM Fla was synthesized and characterized (Figures S7–S9) and was then loaded into the hollow mesoporous MnO_2_ (MnO_2_/Fla). The Fla-loading capacities reached a rather high value of 26% determined by ultraviolet-visible (UV-vis) spectra (Figure S10). The fluorescence spectra also suggested the successful embedment of Fla into MnO_2_ (Figures S11 and S12). These results demonstrated that Fla was stably embedded into the hollow mesoporous MnO_2_.

Then the mature neutrophils were isolated from the rat bone marrow and the purity was measured to be higher than 90% (Figure 2G). LPS was chosen to activate neutrophils in response to inflammatory cues. CD11b/c, neutrophil-specific surface proteins, are upregulated on the occurrence of inflammation and facilitates neutrophil aggregation, migration, and adhesion to substrates by opsonization, chemotaxis (Orden et al., 2014; Diamond and Springer, 1993). As expected, the CD11b/c expression level of neutrophils dramatically increased after treatment with LPS, which confirmed the activation of neutrophils (Figure 2H). Then membrane derived from the purified and activated rat bone marrow neutrophils was cloaked on MnO_2_/Fla (Neu-MnO_2_/Fla) by previous methods (Zhang et al., 2018). The Neu-MnO_2_/Fla exhibited obvious neutrophil membrane coating by transmission electron microscopy (TEM) (Figure 2D). In addition, the surface zeta potential of Neu-MnO_2_/Fla was less negative than that of MnO_2_ and matched with neutrophil membrane-derived vesicles (Figure 2E). Furthermore, flow cytometry measurements confirmed the presence of neutrophil-specific surface protein CD11b/c on Neu-MnO_2_/Fla (Figure 2H). These results further demonstrated the successful coating of neutrophil membrane. Besides, Neu-MnO_2_/Fla showed <10% of Fla release in different solutions for the whole experiment period (Figure S13A) and could stably disperse in different solutions (Figures S13B and S14). These results indicated that Neu-MnO_2_/Fla was highly stable in different solutions under our experimental conditions. In addition, as shown in Figure S15, Fla was almost completely decomposed under white light within 7 days. Thus, the Fla and Neu-MnO_2_/Fla should be protected from prolonged exposure to white light.

**Figure 3:**
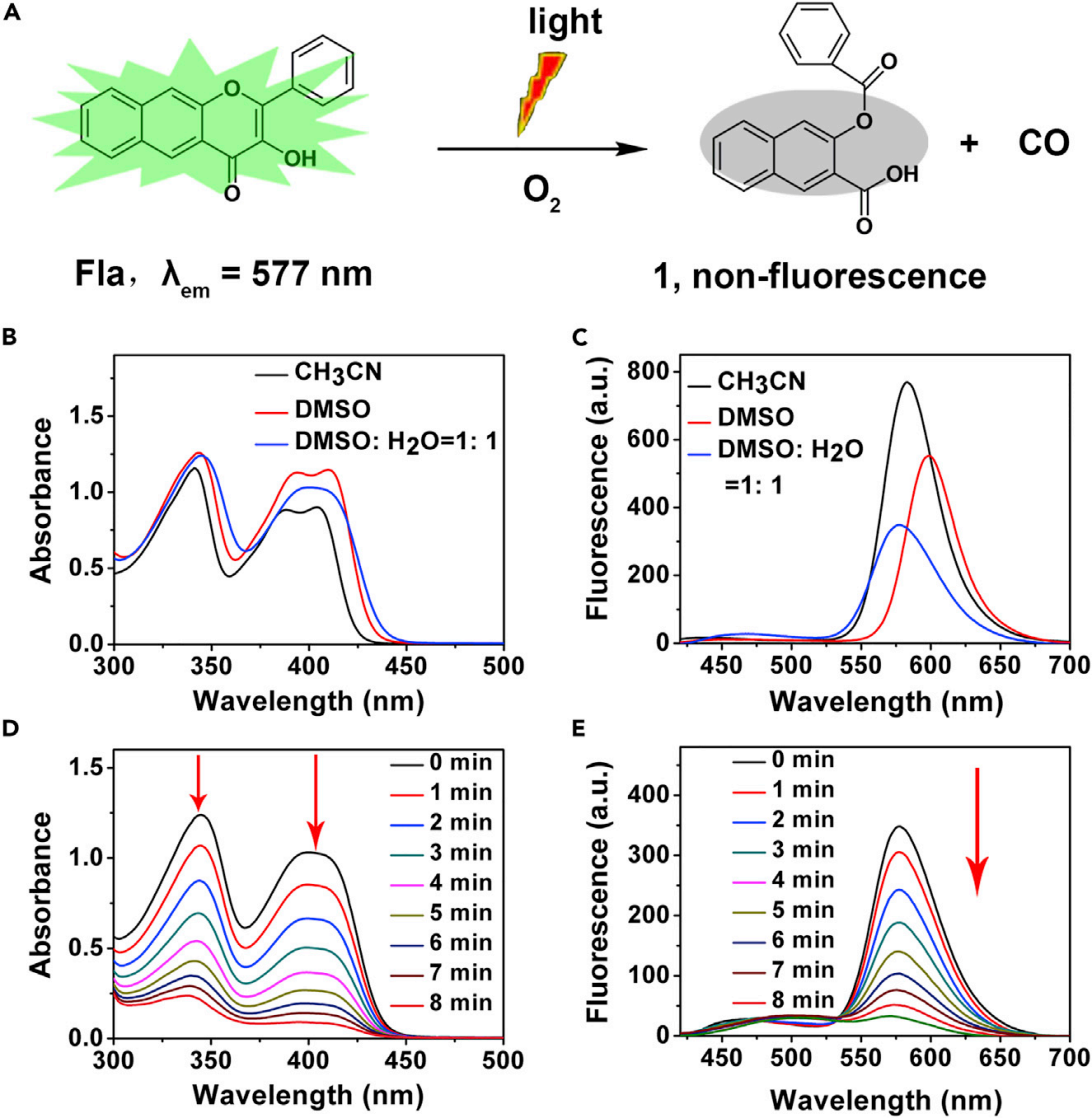
Photo-Induced CO Release Reaction of Fla (A) Schematic illustration of photo-induced CO release reaction of Fla. (B and C) (B) Absorption and (C) Emission spectra of Fla (0.1 mM) in various solvents (λex = 409 nm). (D and E) (D) Absorption and (E) Emission spectra of Fla (0.1 mM) with the light illumination (λ = 410 nm; power = 15 mW/cm^2^) under air for 8 min at 37°C in DMSO: H_2_O (1:1, v/v).

As shown in Figure 3A, Fla could undergo a photo-induced CO release reaction in aerobic environment accompanied with generation of non-emissive 3-(benzoyloxy)-2-naphthoic acid (marked as **1**, Figures S16 and 17). The absorption and emission features of Fla in different solutions were distinct indicating that the CO release was identifiable by either absorption or emission spectroscopy (Figures 3B and 3C). The absorption and emission features of Fla at concentration of biological experiments (1% DMSO, v/v) were also observed (Figure S18). The conversion of Fla to compound 1 would lead to the release of CO and the disappearance of absorption and emission features (Soboleva et al., 2017), so it was feasible to achieve “real-time” monitoring of CO release by spectral changes. Subsequently, the capability of photo-induced CO release of Fla in DMSO: H_2_O (1: 1, v/v) was performed during light illumination (λ = 410 nm; power = 15 mW/cm^2^). Gradually decreasing of the absorbance and fluorescence was observed upon increasing of the illumination time within 8 min (Figures 3D and 3E). In view of the photolysis products **1** possessing no corresponding absorption and emission features, these results suggested the effective occurrence of CO release reaction under our experimental conditions. Moreover, we also measured the changes in the absorption and emission spectra of Neu-MnO_2_/Fla upon illumination (Figure S19). Subsequently, the CO release of Neu-MnO_2_/Fla in a mixed solution (DMSO/PBS, pH 5.5) was investigated by gas chromatography-mass spectrometry (GC-MS). Figure S20 indicated that our design had effective and reliable CO-producing ability. Based on the analysis of GC-MS, 14.7 ppm of CO was released from the Neu-MnO_2_/Fla. By calculating, approximately 58% of Fla incorporated into the MnO_2_ converted to CO. The quantum yield was measured to be 0.006 by the absolute quantum yield measurement system.

**Figure 4:**
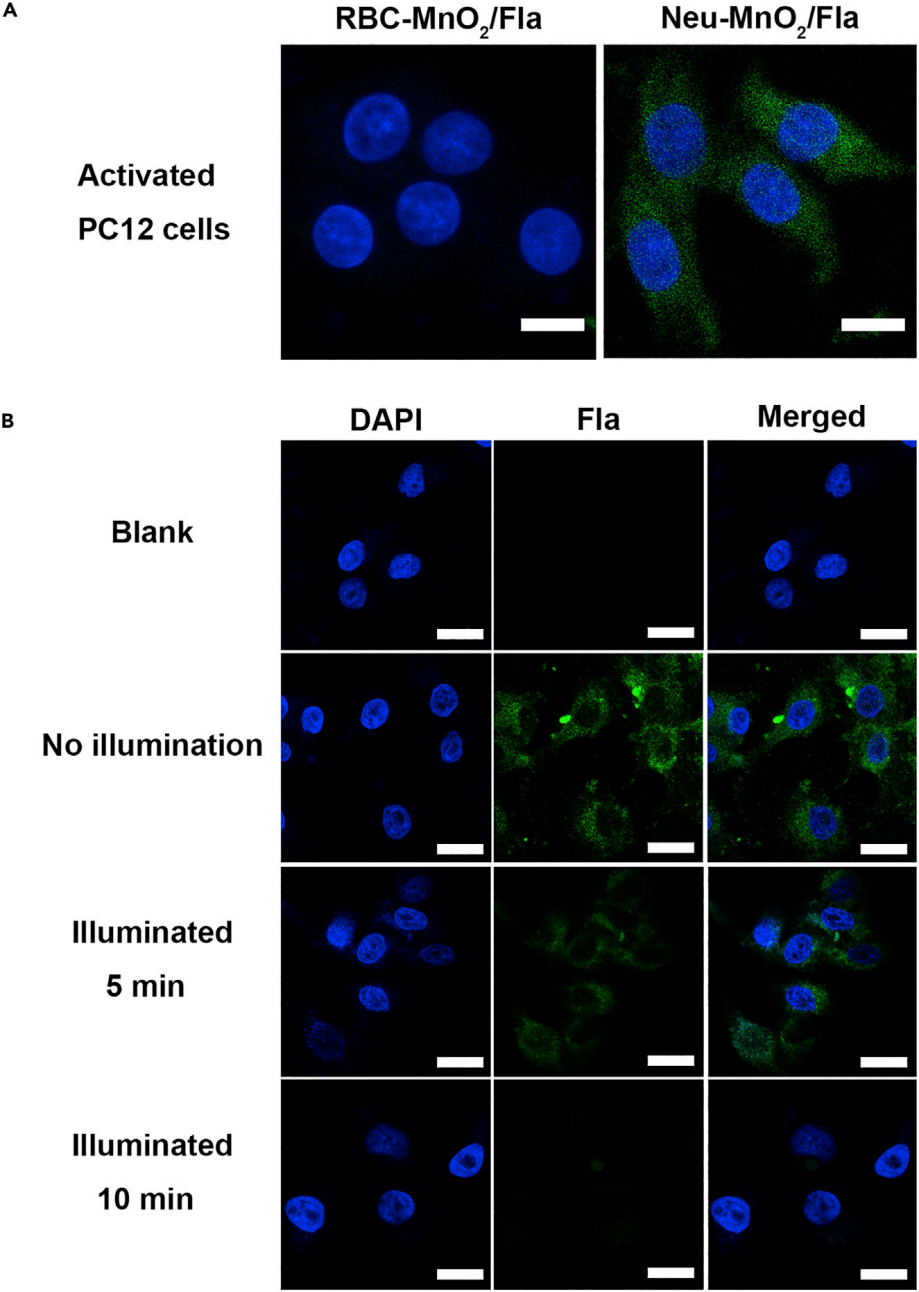
The Inflammation Targeting Ability and Intracellular CO Delivery of Neu-MnO2/Fla (A) Fluorescent images of PC12 cells after incubation with RBC-MnO2/Fla (25 μg/mL) and Neu-MnO2/Fla (25 μg/mL), respectively. Cells were activated with LPS before being treated with nanoparticles. Scale bars: 10 μM. (B) Fluorescent images of PC12 cells exposed to Neu-MnO2/Fla (25 μg/mL) with subsequent illumination. Blue and green represented DAPI and Fla fluorescence, respectively. Scale bars: 25 μM.

Thereafter, the inflammation targeting ability and intracellular CO delivery of Neu-MnO_2_/Fla were investigated. Before cell-based studies, we first tested the cytotoxicity of Neu-MnO_2_/Fla in the presence/absence of illumination. MTT assays showed low toxicity of Neu-MnO_2_/Fla toward PC12 cells under non-illuminated or illuminated conditions at our experimental concentration (Figures S21 and S22). We next comparatively examined the inflammation targeting ability of Neu-MnO_2_/Fla. Neutrophil membrane coating endowed the Neu-MnO_2_/Fla with the specific inflammation targeting ability (Zhang et al., 2019). Herein, Neu-MnO_2_/Fla was added to PC12 cells activated with LPS. Red blood cell membrane-coated MnO_2_/Fla (RBC-MnO_2_/Fla) was used as the control because RBC-MnO_2_/Fla had analogous structures as Neu-MnO_2_/Fla but lacked the inflammation targeting ability. In contrast to RBC-MnO_2_/Fla, the fluorescence of Fla was much stronger for cells treated with Neu-MnO_2_/Fla (Figures 4A and S23). These results demonstrated that the Neu-MnO_2_/Fla successfully targeted to inflamed cells by the neutrophil membrane coating. The protein-receptor interactions mediated by CD11b/c on the neutrophil membrane may play a key role (Orden et al., 2014; Diamond and Springer, 1993; Weaver et al., 2000). The drug release behaviors of Fla from Neu-MnO_2_/Fla was then measured in PBS at different pH values (Figure S24). Compared with the release curve in acidic solution (pH = 5.5), the release amount of Fla was much higher than that in the physiological solution (pH = 7.4) after 24 h, owing to the acidic triggered decomposition of MnO_2_ nanoshells. We further examined the intracellular CO release of Neu-MnO_2_/Fla by monitoring the green fluorescence of Fla. PC12 cells were incubated with Neu-MnO_2_/Fla for 8 h. After washing with PBS, cells were illuminated (λ = 410 nm; power = 15 mW/cm^2^) for different periods of time. Importantly, the green fluorescence was gradually attenuated over illumination time (Figures 4B and S25). These results demonstrated that Neu-MnO_2_/Fla could responsively release CO in cells.

**Figure 5:**
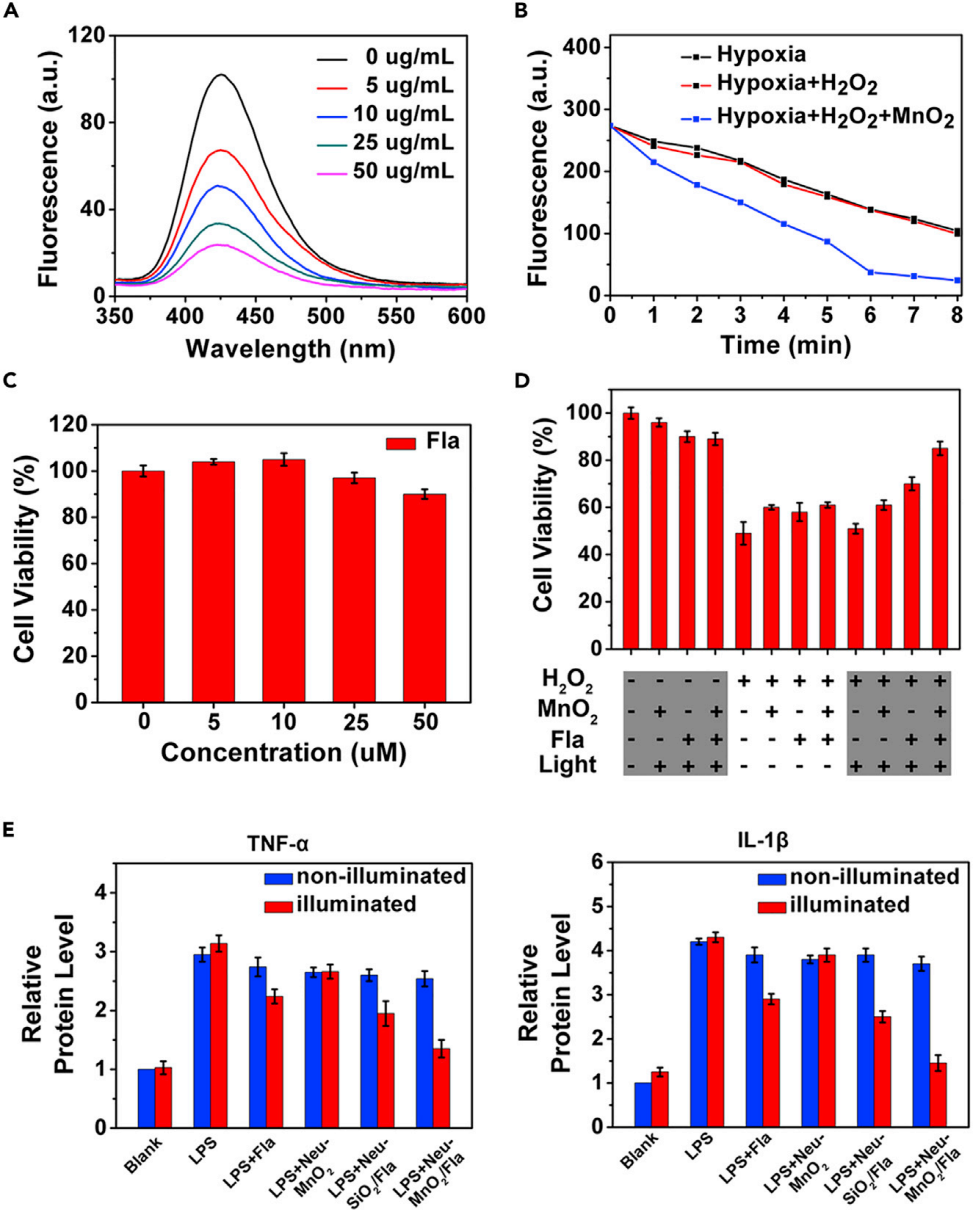
The *In Vitro* Synergistic Anti-inflammatory Effects of Neu-MnO2/Fla (A) CAT-like activity of MnO_2_ with different concentrations. (B) Linear curves of photo-induced emission changes of Fla under hypoxic conditions with MnO_2_ boost (λ = 410 nm; power = 15 mW/cm^2^). (C) PC12 cell viability after incubation with different concentrations of Fla for 24 h. (D) MTT assays of the synergistic antioxidative effects in PC12 cells. (E) Anti-inflammatory effects of the Neu-MnO_2_/Fla in PC12 cells under illuminated and non-illuminated conditions (λ = 410 nm; power = 15 mW/cm^2^).

Having demonstrated the inflammation targeting ability and intracellular CO release of Neu-MnO_2_/Fla, we further studied the synergistic anti-inflammatory effects of Neu-MnO_2_/Fla. The catalase (CAT)-like activity of MnO_2_ was first investigated via a terephthalic acid (TA) reaction (Figure 5A) (Li et al., 2017). In addition, the concentration of H_2_O_2_ was directly evaluated via UV-vis spectroscopy according to the absorbance at 240 nm (Figure S26). Coinciding with the results of TA reaction assay, H_2_O_2_ could be decomposed with the assistance of MnO_2_ in a dose-dependent manner (Figure S27). The concentration of H_2_O_2_ sharply decreased along with the reaction time (Figure S28). Then, we examined the photo-induced CO release under hypoxic conditions with MnO_2_ boost. Illumination of Fla (0.1 mM) in DMSO:H_2_O (1: 1, v/v) under hypoxic conditions resulted in 49% conversion to CO within 6 min. Addition of H_2_O_2_ (1 mM) and MnO_2_ (5 µg/mL) to the solution boosted this reaction, which reached >90% completion within 6 min (Figures 5B and S29). These results indicated that the oxygen from MnO_2_-catalyzed H_2_O_2_ decomposition remarkably boosted the CO release reactivity of Fla. Given the fluctuations of H_2_O_2_ concentrations within inflammatory cells, we performed an additional experiment with 25 μM Fla and 25 μM H_2_O_2_ under hypoxic conditions. Figure S30 showed that CO release was also almost completed within 6 min. This result indicated that, under low concentration of H_2_O_2_ (25 μM), the O_2_ produced from the nanozymes catalysis was sufficient to promote the CO release in physiological conditions. As reported, aberrant reactive oxygen species (ROS) generation was a key mediator during the inflammatory process (Yao et al., 2018b; Yang et al., 2019; Zhang and Kaufman, 2008; Huang et al., 2016). Thus, we further examined the synergistic antioxidative effects of MnO_2_ nanozymes and Fla-generated CO in PC12 cells. First, MTT assays showed low toxicity of MnO_2_ and Fla toward PC12 cells at our experimental concentration (Figures 5C and S31). As shown in Figure 5D, H_2_O_2_ (500 μM) was employed as an inducer of oxidative stress in PC12 cells. Compared with the control without H_2_O_2_ treatment, cytotoxic H_2_O_2_ obviously caused cell death; however, the individual MnO_2_ (25 μg/mL) and Fla-generated CO both improved the cell viability, respectively. As expected, even better effect was observed for cells when treated together with MnO_2_ (25 μg/mL) and Fla (25 μM), verifying the synergistic antioxidative effect of Neu-MnO_2_/Fla.

Afterward, we examined the anti-inflammatory effects of Neu-MnO_2_/Fla against LPS-induced inflammation in PC12 cells. TNF-α and IL-1β, typical pro-inflammatory cytokines, were selected to verify the inflammatory response of PC12 cells. As shown in Figure 5E, PC12 cells displayed a strong inflammatory response after stimulation with LPS as the levels of TNF-α and I`L-1β were significantly increased. In the absence of illumination, all the treatments had no obvious effects on these pro-inflammatory cytokines (blue bars). Illumination with the treatment of Neu-MnO_2_ (without Fla) also did not lead to obvious influence of these pro-inflammatory cytokines. When PC12 cells were treated with Neu-MnO_2_/Fla and light illumination (λ = 410 nm; power = 15 mW/cm^2^), this caused significant inhibition of the expression of TNF-α and IL-1β. The anti-inflammatory effect of Neu-MnO_2_/Fla was better than that of Neu-SiO_2_/Fla. This result indicated that the nanozyme activity of MnO_2_ did help to improve the synergistic effect of Neu-MnO_2_/Fla.

**Figure 6:**
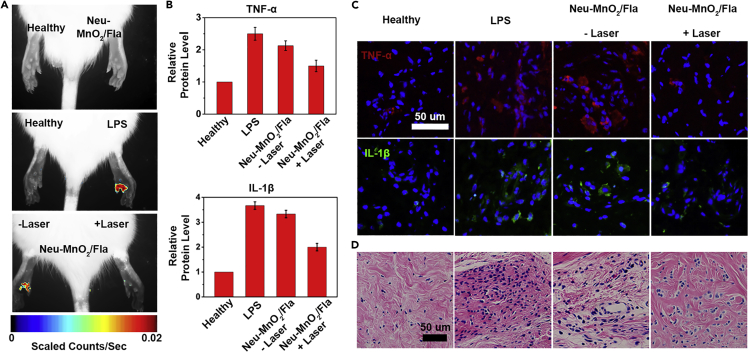
The *In Vivo* Synergistic Anti-inflammatory Effects of Neu-MnO2/Fla (A) *In vivo* imaging of ROS in LPS-induced inflamed paws following treatment with Neu-MnO_2_/Fla (25 μg/mL) without/with two-photon laser irradiation (λ = 820 nm, using confocal laser source, 4,100 mW/cm^2^ at 4% laser power). (B–D) (B) Levels of inflammatory cytokines TNF-α and IL-1β and (C) corresponding fluorescence images and (D) H&E-stained images of inflamed paw.

Then, the *in vivo* anti-inflammatory effects of Neu-MnO_2_/Fla were investigated by using an LPS-induced paw inflammation model following the published study with minor modification (Wan et al., 2017). Owing to the low phototoxicity and deep tissue penetration of two-photon technology, a two-photon approach was used to trigger CO release from Neu-MnO_2_/Fla (Li et al., 2018b). First, the *in vivo* ROS scavenging capability of Neu-MnO_2_/Fla was studied. Levels of ROS in inflamed paws were measured using the *in vivo* imaging system. As demonstrated in Figure 6A, there was no obvious fluorescence in the healthy paw or that treated with the Neu-MnO_2_/Fla alone. In contrast, a strong fluorescence was observed in the LPS-treated paw, confirming the production of excess ROS in the LPS-induced inflamed tissues. When treated with Neu-MnO_2_/Fla and the radiation (marked as Neu-MnO_2_/Fla + Laser), the fluorescence intensity of the inflamed paw was significantly weaker than that of the inflamed paw without laser illumination (marked as Neu-MnO_2_/Fla - Laser), indicating laser illumination decreases the ROS level in the inflamed tissues. Treatment with Neu-MnO_2_/Fla + Laser also significantly reduced the TNF-α and IL-1β levels in the inflamed tissues (Figures 6B and 6C). Hematoxylin and eosin (H&E)-stained images showed that treatment with Neu-MnO_2_/Fla + Laser reduced infiltration of the inflammatory cells (Figure 6D). These results indicated that Neu-MnO_2_/Fla produced a high enough local therapeutic concentration of CO and exhibited a significant anti-inflammatory effect.

### Conclusions

In summary, using a photo-induced CORM and neutrophil membrane coated hollow mesoporous MnO_2_ nanoparticles as carriers and nanozymes, we successfully constructed a spatiotemporally controllable CO-releasing platform for synergistic anti-inflammation in biological system. This approach could deliver CO to the desired location in a safe and effective way. Importantly, remarkable synergistic anti-inflammatory effects were achieved through combining MnO_2_ nanozymes and CO gas therapy. The Neu-MnO_2_/Fla reduced the level of ROS and pro-inflammatory cytokines both *in vitro* and *in vivo*. Histological examinations of tissue sections confirmed the ability of Neu-MnO_2_/Fla to mitigate tissue inflammation. Our work may promote controllable CO-based targeting gas therapy for *in vivo* synergistic anti-inflammation.

### Limitations of the Study

We constructed a spatiotemporally controllable CO-releasing platform for CO gas therapy, and a photo-induced CORM, Fla, was the CO donor. Fla was known to undergo CO release via direct illumination using visible light, but the visible light limited a lot of applications of CO gas therapy. Although Fla could be effectively excited by two-photon laser, two photon experiment had certain requirements for equipment and operation. Moreover, the low fluorescence quantum yield of Fla may affect the amount of CO released in biological tissues. Therefore, it is necessary to develop near-infrared light-induced CORM with high fluorescence quantum yield to broaden the applications of CO gas therapy.

### Resource Availability

#### Lead Contact

Further information and requests for resources and reagents should be directed to and will be fulfilled by the Lead Contact, Xiaogang Qu (xqu@ciac.ac.cn).

#### Materials Availability

This study did not generate new unique reagents.

#### Data and Code Availability

The published article includes all datasets generated or analyzed during this study.

## Methods

All methods can be found in the accompanying Transparent Methods supplemental file.

## Supplemental Information

Document S1. Transparent Methods and Figures S1–S31
