# Supplementary material for: Carbon Monoxide Controllable Targeted Gas Therapy for Synergistic Anti-inflammation
Source: iScience. 2020 Aug 20;23(9):101483. doi: 10.1016/j.isci.2020.101483 (PMC7479631; doi:10.1016/j.isci.2020.101483)
Supplement: Document S1. Transparent Methods and Figures S1–S31 [file mmc1.pdf]

iScience, Volume *23*

Supplemental Information

Carbon Monoxide Controllable Targeted Gas Therapy for Synergistic Anti-inflammation

Chun Liu, Zhi Du, Mengmeng Ma, Yuhuan Sun, Jinsong Ren, and Xiaogang Qu

Supplemental figures

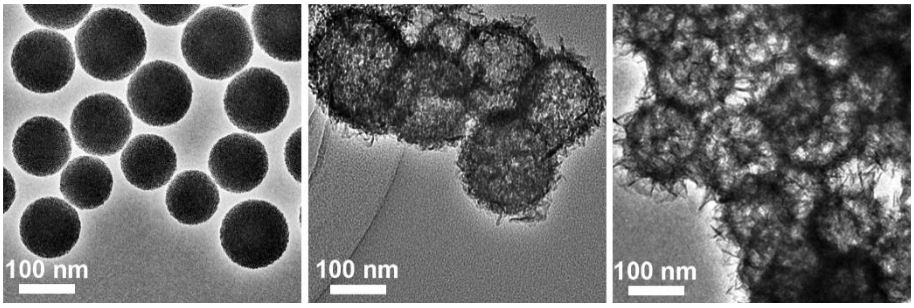

Figure S1. Electron microscopic characterization, Related to Figure 2. Transmission electron microscopy (TEM) images of SiO_2, $SiO_2@MnO_2$, and MnO_2, Related to Figure 2.

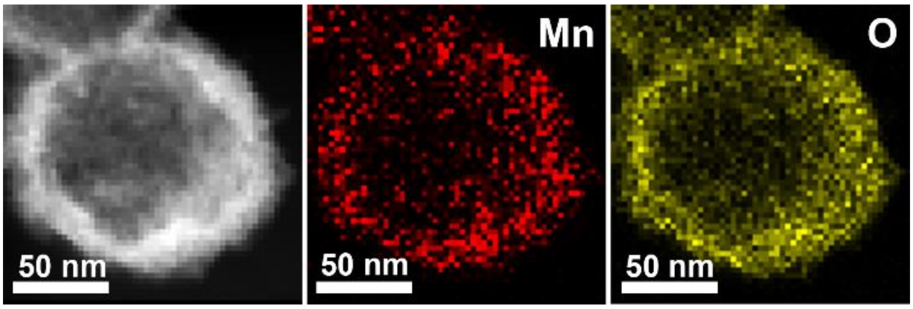

Figure S2. Elemental mapping for MnO_2, Related to Figure 2.

The high-angle annular dark-field scanning TEM-based elemental mapping for MnO_2.

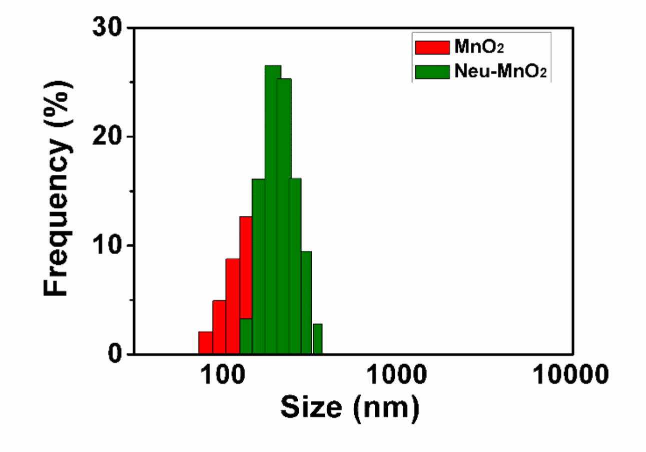

Figure S3. DLS measurements, Related to Figure 2.

Hydrodynamic diameter of MnO_2 and Neu-MnO_2 by DLS measurements. The average hydrodynamic diameters of Nue-MnO_2 increased from 188 nm to 244 nm after the coating of neutrophils membrane.

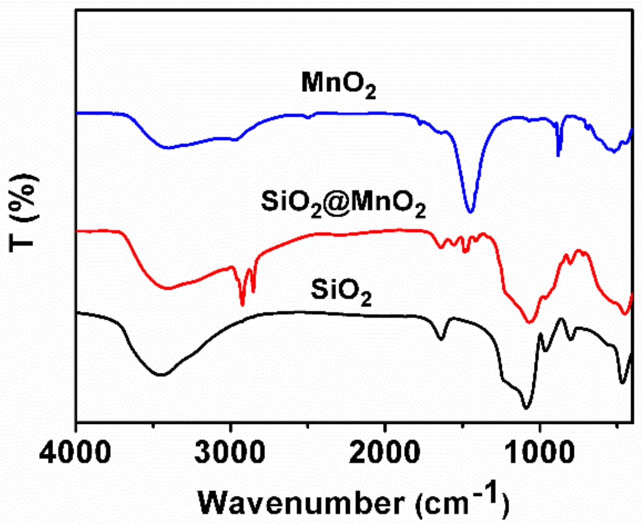

Figure S4. Fourier transform infrared spectroscopy (FTIR) analysis, Related to Figure 2.

Fourier transform infrared spectroscopy (FTIR) of SiO_2, SiO_2@MnO_2, and MnO_2. FT-IR spectra of SiO_2, SiO_2@MnO_2 showed characteristic peaks in the region of 798 cm^{-1} and 470 cm^{-1} arising from the stretching vibration of the Si-O and Si-O-Si bonds. FT-IR spectra of MnO_2 had characteristic peak in the region of 520 cm^{-1} arising from the stretching vibration of the Mn-O bonds. From the FT-IR spectra of MnO_2, the disappearance of characteristic peak of SiO_2 and the existence of characteristic peak of MnO_2 indicated that the silica were etched and the MnO_2 were prepared.

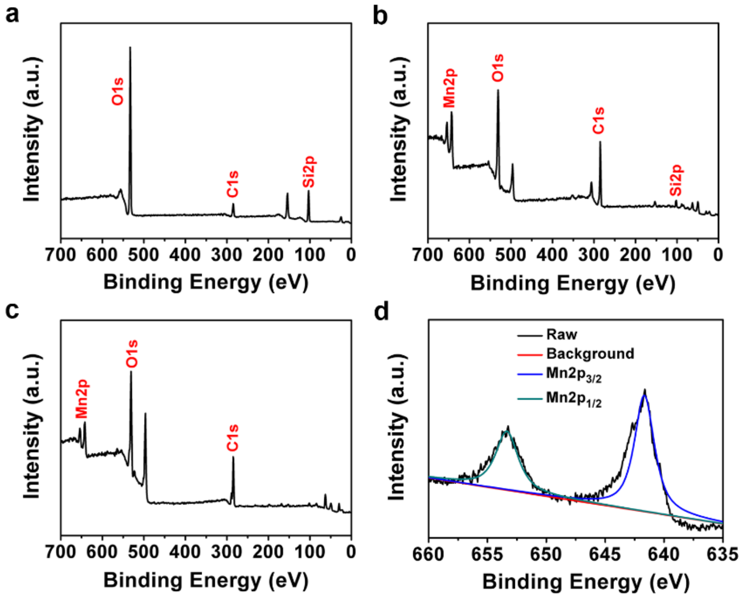

Figure S5. X-ray photoelectron spectroscopy (XPS) analysis, Related to Figure 2.

X-ray photoelectron spectroscopy (XPS) analysis of a) SiO_2, b) SiO_2@ MnO_2, c) MnO_2 and d) fitted $Mn2p_{1/2}$ and $Mn2p_{3/2}$ photoelectron peaks of MnO_2. The binding energy (BE) values of $Mn2p_{3/2}$ and $Mn2p_{1/2}$, 641.7 eV and 653.39 eV, respectively, correspond to the MnO_2. The disappearance of the peak of SiO_2 and existence of the peak of MnO_2 in **Figure S5**c indicated that the silica were etched and the MnO_2 were prepared.

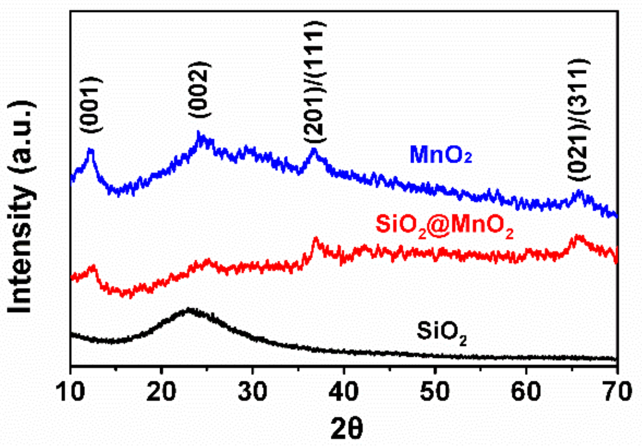

Figure S6. The X-ray diffraction (XRD) analysis, Related to Figure 2.

The X-ray diffraction (XRD) patterns of SiO_2, SiO_2@ MnO_2, and MnO_2. XRD patterns showed that the diffraction peaks could match well with the crystal phase of δ-MnO_2 (JCPDS No. 80-1098).

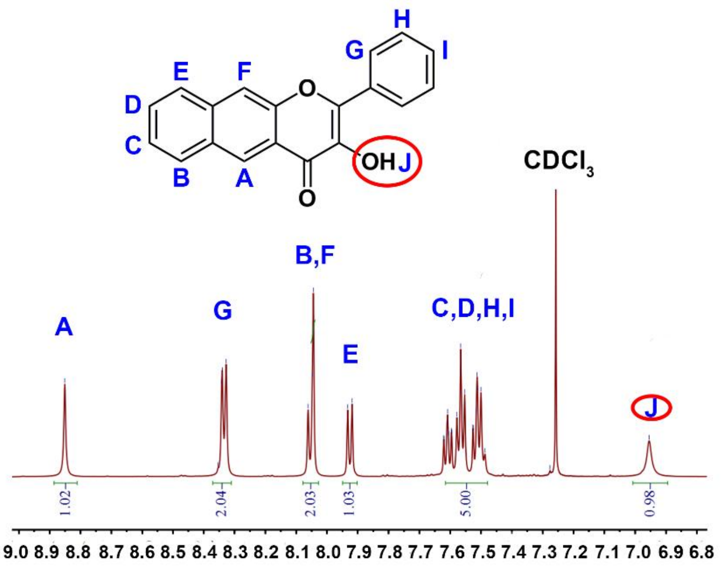

Figure S7. ^{1}H NMR spectrum of Fla in $CDCl_3$, Related to Figure 3.

^{1}H NMR (600 MHz), δ (ppm): 8.88 (s, 1H), 8.35 (d, J = 5.4 Hz, 2H), 8.08 (d, s, J = 3.6 Hz, 2H), 7.95 (d, J = 6.3 Hz, 1H), 7.67-7.45 (m, 5H), 6.93 (s, 1H).

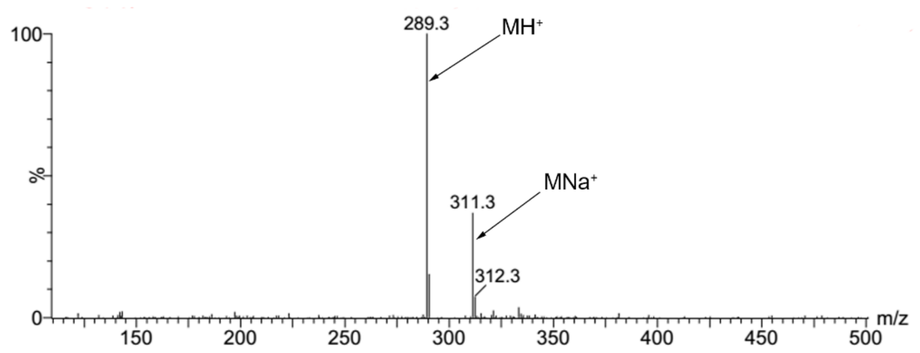

Figure S8. ESI-MS of Fla, Related to Figure 3.

Anal. calcd for $C_{19}H_{13}O_3$ $[MH]^+$: 289.1; found: 289.3.

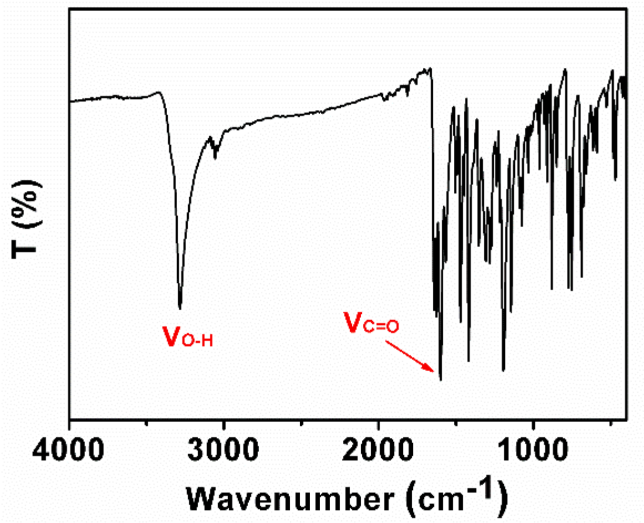

Figure S9. FTIR spectrum of Fla, Related to Figure 3.

The peak at 1596 cm^{-1} was attributed to the stretching of C=O and the peak at 3290 cm^{-1} was attributed to the stretching and bending vibration of O-H.

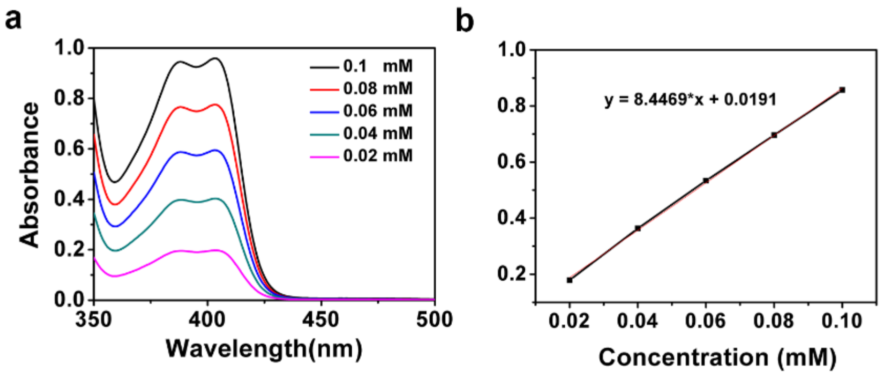

Figure S10. Absorbance measurement of Fla in CH_3CN of various concentrations, Related to Figure 3.

a) Absorption spectra of Fla in CH_3CN of various concentrations. b) Fla-concentration dependent calibration curve of the absorbance at 409 nm.

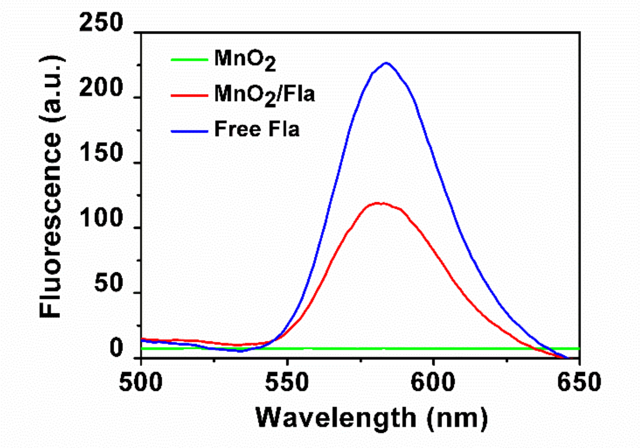

Figure S11. Fluorescence spectra measurement, Related to Figure 3.

Fluorescence spectra of MnO_2 (25 ug/mL), MnO_2/Fla (25 ug/mL), and free Fla (25 uM) in CH_3CN (λ_{ex} = 409 nm). The emission characteristic peak of Fla was persisted in the spectra of MnO_2/Fla with slightly shift, suggesting the successful embedment of Fla into MnO_2. The slight shift in the fluorescent emission observed for Fla upon incorporation into the MnO_2 nanomaterial could be the influence of the absorption of MnO_2. In a word, the nature and function of Fla was not changed after incorporation into the MnO_2 nanomaterial.

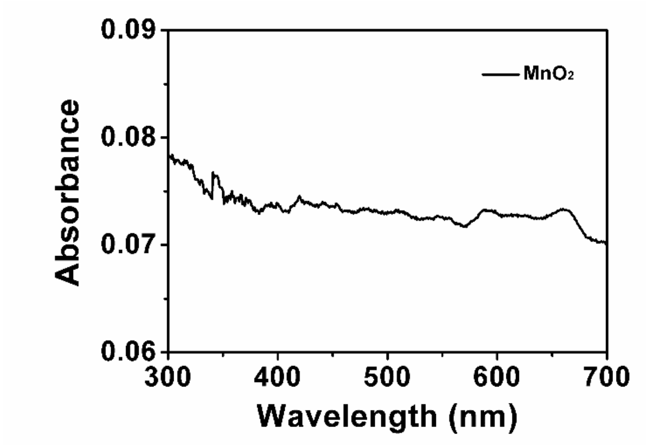

Figure S12. Absorbance measurement of MnO_2, Related to Figure 2.

Absorption spectra of MnO_2 (25 ug/mL) in CH_3CN. The MnO_2 showed a broad range of absorption in the UV-vis regions.

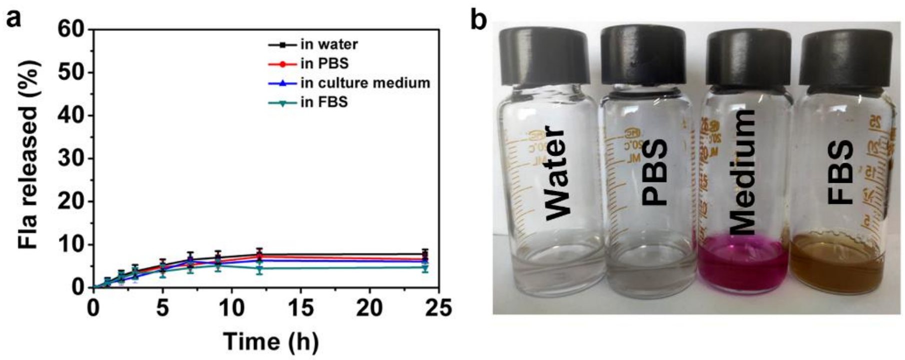

Figure S13. Stability of Neu-MnO_2/Fla in different solutions, Related to Transparent Methods and Figure 2.

a) Fla release behaviors of Neu-MnO_2/Fla in different solutions within 24 hours; The amount of released drug was determined by measuring the absorbance of the drug from the supernatant liquid at different time point, quantified from a standard curve. b) Photos of Neu-MnO_2/Fla (25 ug/mL) in different solutions.

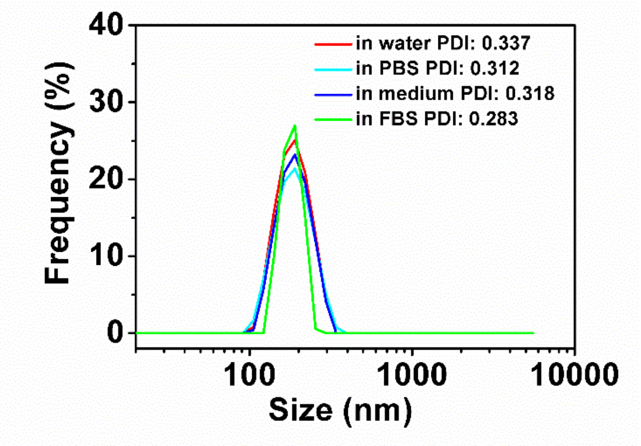

Figure S14. Stability of Neu-MnO_2/Fla in different solutions, Related to Figure 2. The DLS measurements and PDI index of Neu-MnO_2/Fla (25 ug/mL) in different solutions.

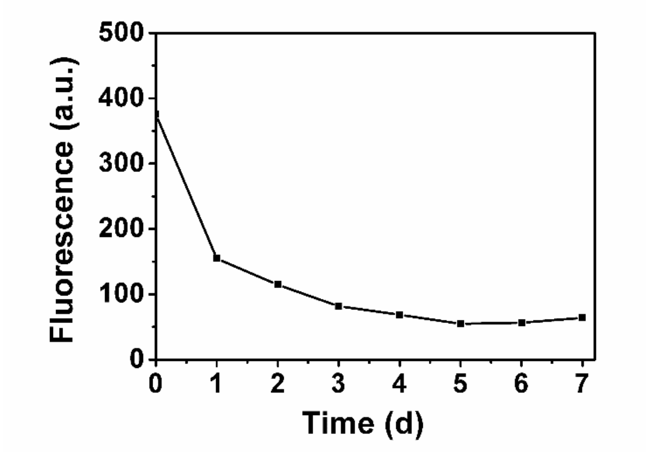

Figure S15. Photostability of Fla under white light, Related to Figure 3.

Fluorescence emission changes of Fla (0.1 mM) under white light in DMSO: H_2O = 1: 1 within 7 days. The fluorescence emission was monitored at 576 nm.

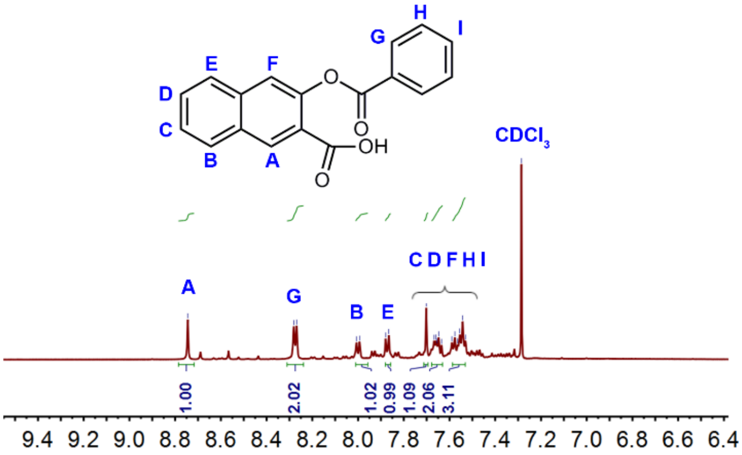

Figure S16. ^{1}H NMR spectrum of 1 in $CDCl_3$, Related to Figure 3.

^{1}H NMR ($CDCl_3$, 600 MHz), δ (ppm): 8.74 (s, 1H), 8.26 (d, 2H), 7.99 (d, 1H), 7.86 (d, 1H), 7.72-7.46(m, 6H) ppm.

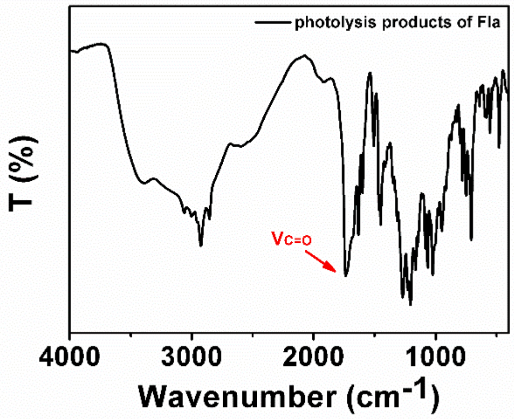

Figure S17. FTIR spectrum of 1, Related to Figure 3.

The peak at 1737 cm^{-1} was attributed to the stretching of C=O.

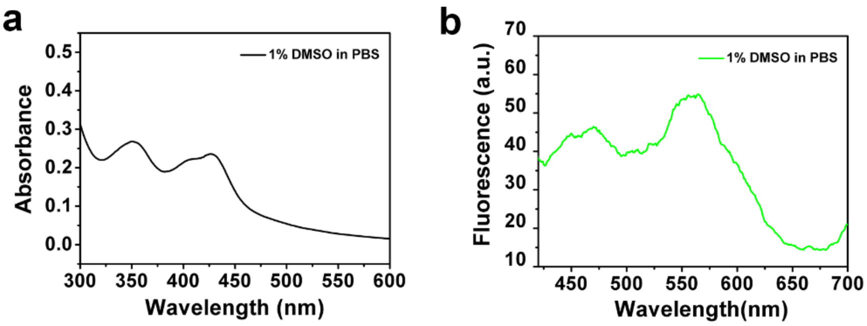

Figure S18. Spectrum measurement of Fla in 1% DMSO in PBS, Related to Figure 3.

a) Absorption and b) Emission spectrum of Fla (25 uM) in 1% DMSO in PBS (λ_{ex} = 409 nm).

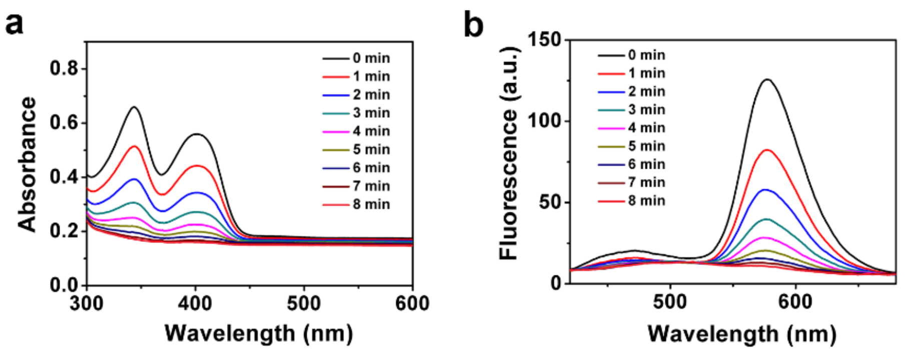

Figure S19. Photo-induced CO release of Neu-MnO_2/Fla, Related to Figure 3.

a) Absorption and b) Emission spectra of Neu-MnO_2/Fla (50 ug/mL) with the light illumination (λ = 410 nm; power = 15 mW/cm^2) under air for 8 min at 37 °C in DMSO: H_2O (1: 1, v/v).

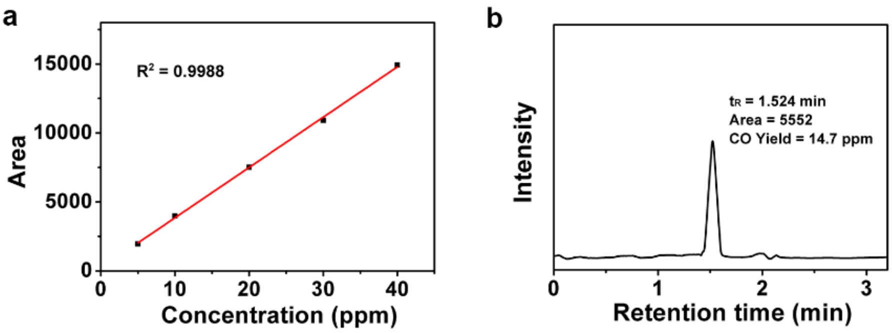

Figure S20. The gas chromatography-mass spectrometry (GC-MS) analysis of CO release from the Neu-MnO_2/Fla, Related to Transparent Methods and Figure 3.

a) GC-MS quantitative analysis standard curve of CO. b) GC-MS chromatogram showing CO at retention time 1.524 min, and the yield was measured to be 14.7 ppm. Experiment details were described in Experimental Procedures.

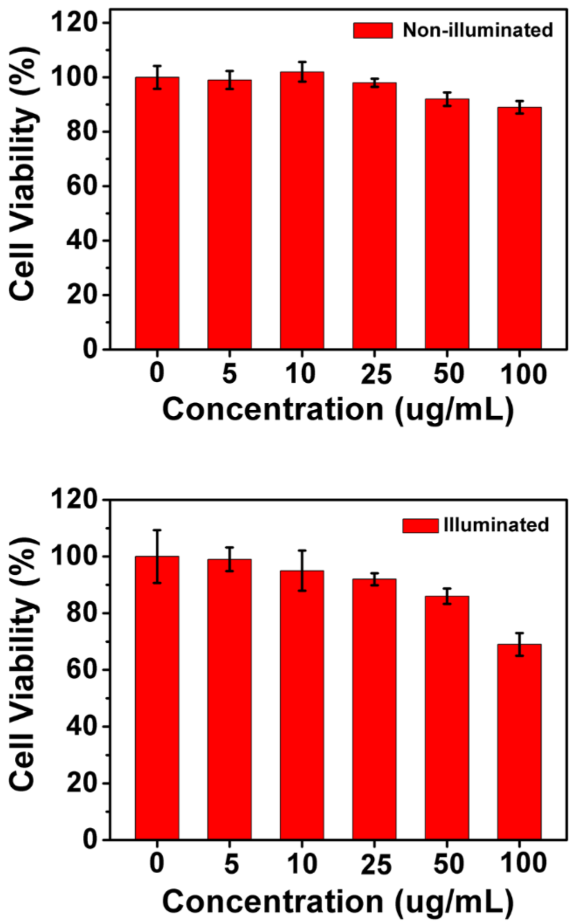

Figure S21. MTT assay of Neu-MnO_2/Fla under different conditions, Related to Figure 4 and Figure 5.

MTT assay of Neu-MnO_2/Fla at different concentrations co-incubated with PC12 cells under non-illuminated or illuminated conditions. Upon light illumination (λ = 410 nm; power = 15 mW/cm^2) to trigger in situ CO release, Neu-MnO_2/Fla also exhibited low cytotoxicity at relatively low experimental concentrations. The modest effect of CO release on cell viability may be due to the tissue protection of CO.

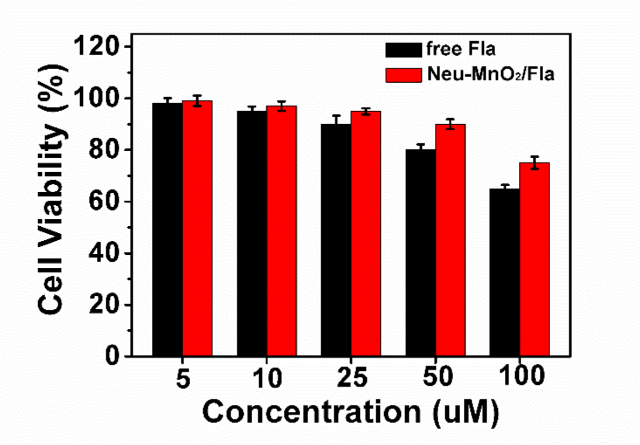

Figure S22. MTT assay of free Fla and Neu-MnO_2/Fla under different conditions, Related to Figure 5.

MTT assay of free Fla and Neu-MnO_2/Fla at different concentrations co-incubated with PC12 cells under illuminated conditions. The concentrations of Neu-MnO_2/Fla were represented by the concentration of Fla loaded into the MnO_2. The CO release from Neu-MnO_2/Fla produced a lower toxic effect than did CO release from free Fla under identical conditions. The lower toxic effect of CO release from Neu-MnO_2/Fla on cell viability may be due to the antioxidant protection of MnO_2 as nanozymes possessing catalase (CAT) activity.

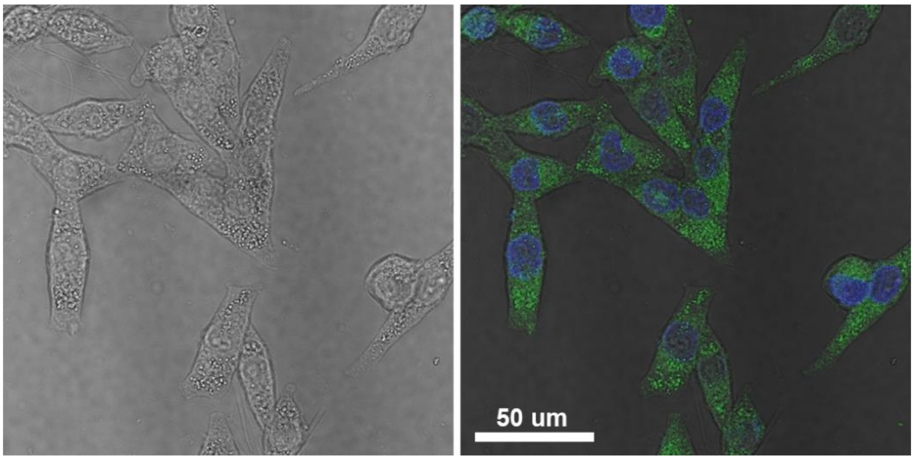

Figure S23. Fluorescence microscopy images, Related to Figure 4.

Fluorescence microscopy images of PC12 cells after incubation with Neu-MnO_2/Fla (25 ug/mL). A bright field image was additionally provided. Cells were activated with LPS before treated with nanoparticles.

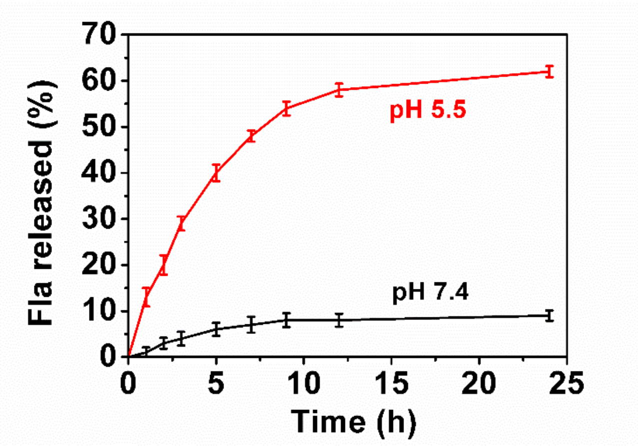

Figure S24. Fla release kinetics measurement, Related to Figure 4.

Fla release kinetics of the Neu-MnO_2/Fla at pH 7.4 and 5.5 in 24 h, respectively. The amount of released drug was determined by measuring the absorbance of the drug from the supernatant liquid at different time point, quantified from a standard curve.

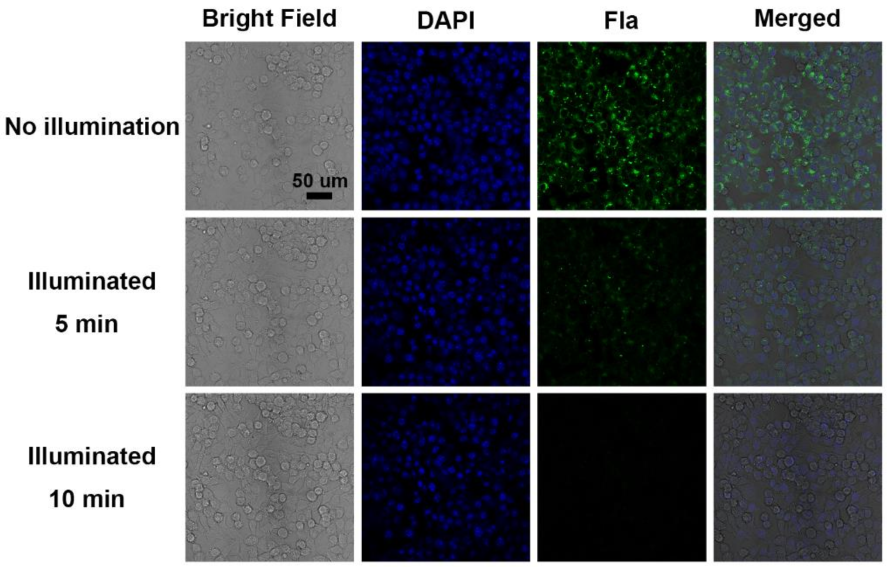

Figure S25. Fluorescent images, Related to Figure 4.

Fluorescent images of PC12 cells exposed to Neu-MnO_2/Fla (25 ug/mL) with subsequent illumination. The same cell regions were given in no illumination, illuminated 5 min and illuminated 10 min for easily evaluating loss of the green emission associated with CO release reactivity. Blue and green represented DAPI and Fla fluorescence, respectively. Scale bars: 50 um. Line 1-3: Cells exposed to Neu-MnO_2/Fla with subsequent illumination for different periods of time resulting in CO release from Fla and loss of emission.

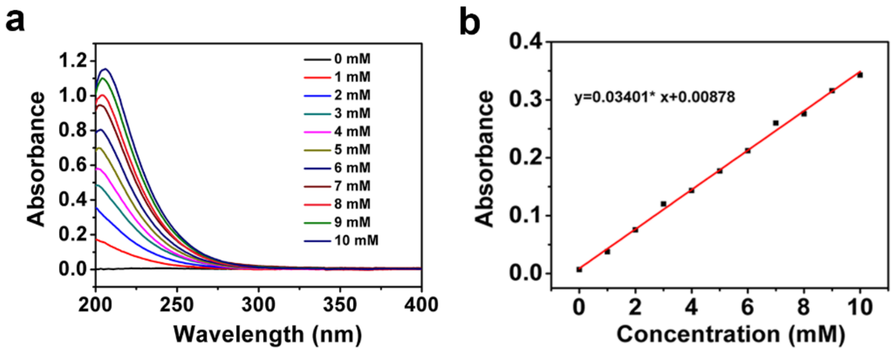

Figure S26. Absorbance measurement of H_2O_2, Related to Figure 5.

a) The UV-vis spectra of H_2O_2 of various concentrations and b) H_2O_2-concentration dependent calibration curve of the absorbance at 240 nm.

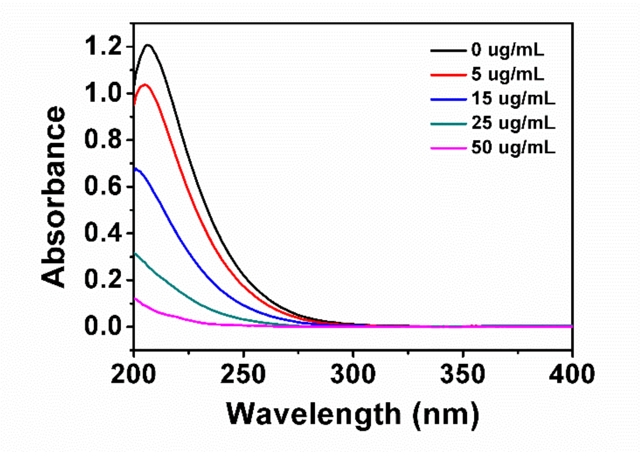

Figure S27. CAT-like activity measurement of MnO_2, Related to Figure 5.

Concentration-dependent decomposition of H_2O_2 (10 mM) with MnO_2.

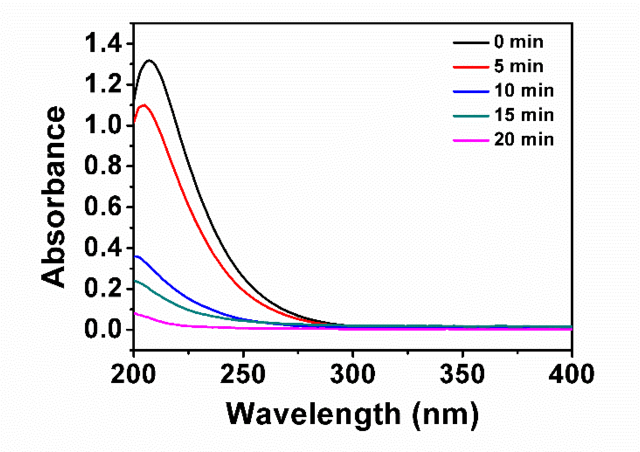

Figure S28. CAT-like activity measurement of MnO_2, Related to Figure 5.

Time dependent decomposition of H_2O_2 (10 mM) upon the treatment of MnO_2 with a concentration of 50 ug/mL.

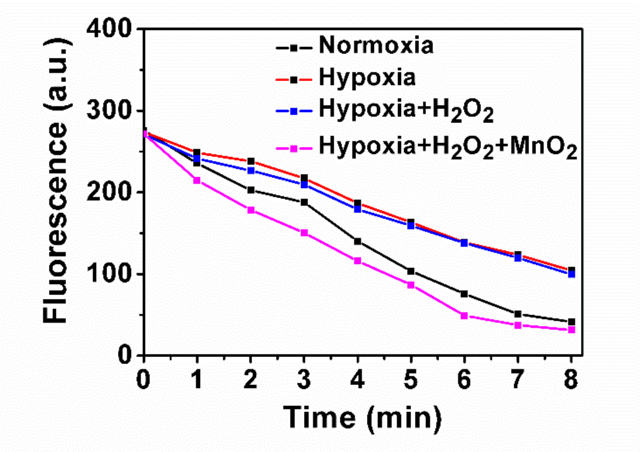

Figure S29. CO release of Fla under different conditions, Related to Figure 5.

Linear curves of photo-induced emission changes of Fla under hypoxic conditions with MnO_2 boost and normoxic conditions (λ = 410 nm; power = 15 mW/cm^2).

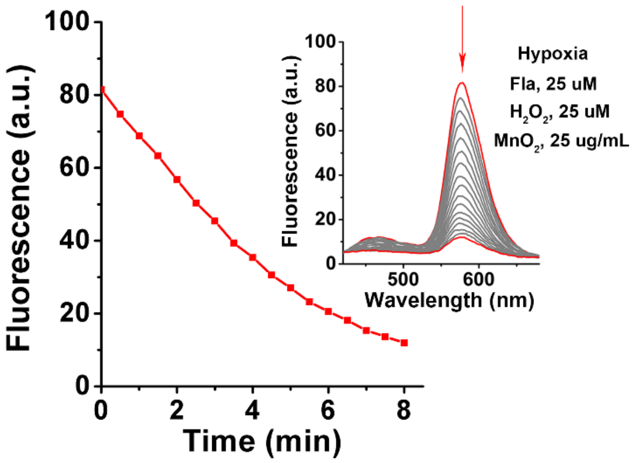

Figure S30. CO release of Fla under hypoxic conditions with MnO_2 boost, Related to Figure 5.

Fluorescence emission changes associated with CO release of Fla (25 uM) under hypoxia + H_2O_2 (25 uM) + MnO_2 (25 ug/mL) (λ_{ex} = 409 nm).

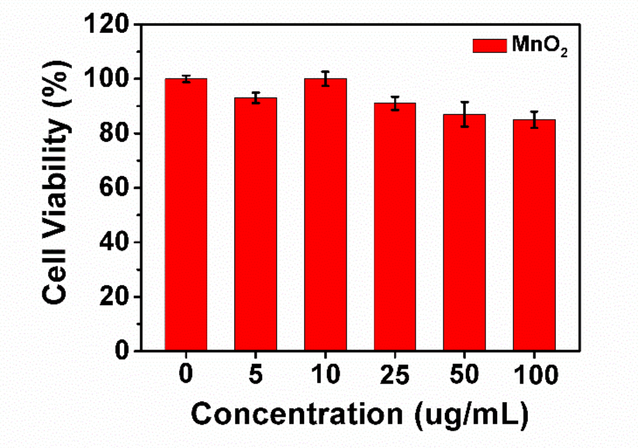

Figure S31. MTT assay of MnO_2, Related to Figure 5.

MTT assay of MnO_2 at different concentrations co-incubated with PC12 cells for 24 hours.

Transparent Methods

Chemicals

Tetraethyl orthosilicate (TEOS), hexadecyltrimethylammonium bromide (CTAB), potassium permanganate ($KMnO_4$), polyvinylpyrrolidone (PVP), sodium hydroxide (NaOH) and aqueous ammonia (28 wt%) were purchased from Sigma-Aldrich. Benzaldehyde, lipopolysaccharide (LPS) were purchased from Sigma-Aldrich. RPMI 1640 medium, Percoll, and Red blood cell lysate were purchased from Solarbio. 1-(3-hydroxynaphthalen-2-yl) was purchased from WuXi Apptec Co.,Ltd. Anti-Ly6g antibody (FITC) (RB6-8C5, ab25024) was purchased from Abcam. PE anti-rat CD11b/c antibody (OX-42) was purchased from BioLegend. Rat TNF-α ELISA kit and rat IL-1β ELISA kit were purchased from mlbio (shanghai).

Apparatus and characterization.

UV-Vis absorbance measurement was carried out on a JASCO V-550 UV-Vis spectrophotometer. Fluorescence spectra was detected by JASCO F-6000 fluorescence spectrometer with a Peltier temperature control accessory. FTIR spectra were carried out on a BRUKE Vertex 70 FT-IR spectrometer. Transmission electron microscopic (TEM) images were captured with a FEI TECNAI G2 20 highresolution transmission electron microscope operating at 200 kV. N_2 adsorption-desorption isotherms were recorded on a Micromeritics ASAP 2020M automated sorption analyzer. The pore size was determined following the BJH method. 410 nm light was obtained through a CEL-HXF300 Xenon lamp with power 15 mW/cm^2. ^{1}H NMR spectrum was recorded on a Bruker-600 MHz NMR instrument. The crystalline structures of the as prepared samples were evaluated by X-ray diffraction (XRD) analysis on a Rigaku-Dmax 2500 diffractometer by using CuKα radiation. X-ray photoelectron Spectroscopy (XPS) spectra were analyzed by Thermo Fisher Scientific ESCALAB 250Xi Spectrometer Electron Spectroscopy (USA). The flow cytometry data was obtained by BD LSRFortessa™ Cell Analyzer.

Synthesis of mesoporous silica nanoparticles.

The mesoporous silica template was synthesized according to a modified Stober method. Briefly, 0.29 g CTAB and 0.10 g PVP were dissolved in 40 mL H_2O in a round bottom flask (250 mL), then 50 mL H_2O, 60 mL methanol and 150 uL aqueous ammonia (28 wt%) were added to the flask and stirred at room temperature. Next, 600 uL TEOS were added and the reaction was kept at room temperature with continuous stirring for 2.5 h. The obtained products were collected by centrifuging and washed by water and ethanol for several times. Finally, the silica nanoparticles were dispersed in 60 mL of acetone and refluxed at 60 °C for 8 h to remove CTAB templates. The obtained sample was washed with ethanol and dried in vacuum at 40 °C for 12 h for further use.

Synthesis of mesoporous hollow MnO_2

The mesoporous hollow MnO_2 was synthesized by a hydrothermal process using the mesoporous silica nanoparticles as templates (Wang et al., 2016). Briefly, 0.20 g mesoporous silica were dispersed in 35 mL H_2O, followed by 1.45 g $KMnO_4$ was added. Then, the mixed solution was transferred into an autoclave, which was then heated at 160 °C for 48 h. The obtained sample was etched with 2 M NaOH solution at 50 °C for 6 h. The obtained sample was washed with ethanol and dried for further use.

Preparation of Fla-loaded MnO_2

At the feeding weight ratio (Fla: MnO_2) of 3: 1, the MnO_2 solution (0.2 mg/mL) was mixed with Fla and the suspension was protected from light and stirred overnight. The as-prepared Fla loaded MnO_2 was collected by centrifugation. Then, the payload of Fla was calculated by measuring the absorbance of the difference between the supernatant after centrifugation and initial solution. The calculated drug-loading capacity of Fla in MnO_2 was about 26%. The release experiment was tested by measuring the absorbance of the supernatant solution at different time point. The amount of adsorbed drug was determined by measuring the absorbance of the drug from the supernatant liquid, quantified from a standard curve. The loading capacity

was calculated as follows:

Loading capacity = $W/(W+W_{MnO2}) \times 100\%$

W is the weight of adsorbed drug in the MnO_2, W_{MnO2} is the weight of total MnO_2.

Isolation of mature rat neutrophil from bone marrow

Neutrophils were isolated from bone marrow of healthy rat following the published protocols with minor modification (Kumar et al., 2010). Briefly, the bones were removed and freed of muscle and sinew. Then bone marrow was flushed from the bones with PBS. The suspension was added slowly into a three-layer Percoll gradient of 55%, 65%, and 78% Percoll, respectively, diluted in PBS (v: v), and centrifuged (500 g, 30 min). As a result, bone marrow cells were layered and the mature neutrophils were recovered at the interface of the 65% and 78% fractions. The obtained neutrophils were identified with anti-Ly6g antibody (FITC) and PE-anti rat CD11b/c antibody (OX-42). The use of antibodies strictly followed the product manual. Ly6g is selectively present at higher levels in neutrophils and is a specifc marker that separates neutrophils from other leukocytes (Mert et al., 2019; Lee et al., 2007). CD11a, CD11b, CD11c are known to be the members of β_2-integrins family of leukocyte membrane glycoproteins, which are widely distributed on neutrophils and lymphocytes for many cell-cell interactions. The neutrophils express three forms of the CD11a, CD11b and CD11c, while lymphocytes express only CD11a (Issekutz and Issekutz, 1993; Pavlovic et al., 1994).

Preparation of cell membrane

Neutrophils membrane was derived following a previously published protocol (Zhang et al., 2018). Briefly, cells were washed with cold PBS and were then suspended in hypotonic lysing buffer containing protease inhibitor. Cells were then disrupted with a homogenizer. The suspension was centrifuged (20000 g, 20 min) at 4 °C. The precipitate was discarded and supernatant was centrifuged again (100000g, 30 min). The RBC membrane was derived following our previous work (Zhang et al., 2018). Membrane was stored at -80 °C for subsequent studies.

Membrane coating

Neutrophil membrane was added to Fla-loaded MnO_2 solution at a particle-to-membrane protein weight ratio of 2:1. Then, the solution was sonicated for 3 min at 4 °C to obtained Neu-MnO_2/Fla. RBC-MnO_2 were obtained following the same procedure.

Fla release behaviors of Neu-MnO_2/Fla in different solutions

The Fla release from Neu-MnO_2/Fla in different solutions was investigated using a UV-Vis spectrophotometer (JASCO V550). 2 mL DMSO solution of the Neu-MnO_2/Fla (5 mg/mL) was respectively dispersed into 18 mL different solutions (including water, PBS, culture medium, serum) under stirring at 37 °C in the capped vials. At predetermined time intervals, 200 µL of the solution was removed for UV-vis spectrum measurement of Fla, quantified from a standard curve.

CO quantization

CO gas was detected and quantified using an Agilent 5975 gas chromatograph-mass spectrometer (GC-MS) with GASPro columns. Neu-MnO_2/Fla nanoparticles (3 mg) was suspended in 3 mL mixed solution (DMSO/PBS, pH 5.5), and the mixed solution was degassed by purging with argon gas. Then, H_2O_2 (1 mM) was added in the solution and the mixed solution was illuminated with 410 nm light for 24 hours. The solution products were quantitatively analyzed by GC-MS through observing and comparing the chromatographic peaks with those for various standards.

CAT-like activity of MnO_2

CAT-like activity was assayed by measuring inhibition of the generation of 2-hydroxyterephthalic acid. Solutions containing terephthalic acid (TA, 0.5 mM), H_2O_2 (10 mM), and MnO_2 of various concentrations were prepared in PBS buffer (pH 7.4, 25 mM). After 24 h of incubation, MnO_2 was centrifuged and the mixtures were measured by using fluorescence analysis (λ_{ex}: 320 nm, λ_{em}: 425 nm).

The concentration of H_2O_2 was evaluated by directly measuring the absorbance at 240 nm via UV-Vis spectrophotometer (JASCO V550). Solutions containing H_2O_2 (10 mM) and MnO_2 of various concentrations were prepared in PBS buffer (pH 7.4, 25 mM). After 20 min of incubation, MnO_2 was centrifuged and the absorbance of the H_2O_2 remaining was measured. In addition, we also explored the time dependent decomposition of H_2O_2 (10 mM) upon the treatment of MnO_2 with a concentration of 50 ug/mL.

CO release of Fla under hypoxic conditions with MnO_2 boost

For the hypoxic condition studies, a solution of DMSO: H_2O (1: 1, v/v) containing Fla (0.1 mM) was degassed by purging with nitrogen gas in a sealed quartz cuvette (total volume 4 mL). The obtained system was illuminated (λ = 410 nm; power = 15 mW/cm^2) over the course of 8 min with an interval of 1 min for periodically measuring emission spectra of the solution mixture. An additional control experiment was performed in the case of hypoxic condition using a solution of DMSO: H_2O (1: 1, v/v) containing Fla (0.1 mM) and H_2O_2 (1 mM).

For experimental group, a solution of DMSO: H_2O (1: 1, v/v) containing Fla (0.1 mM) and H_2O_2 (1 mM) were degassed by purging with nitrogen gas in a sealed quartz cuvette (total volume 4 mL).Then MnO_2 were added immediately at a final concentration of 5 ug/mL. The obtained system was illuminated (λ = 410 nm; power = 15 mW/cm^2) over the course of 8 min with an interval of 1 min for periodically measuring emission spectra of the solution mixture.

Given the concentrations of H_2O_2, Fla and nanoparticles used in subsequent cell studies, we performed an additional experiment with 25 uM Fla and 25 uM H_2O_2 under hypoxic conditions.

Quantum yield measurements

The quantum yield was measured by Absolute Quantum Yield Measurement System (C9920-02, Hamamatsu Photonics K. K., Japan). The sample or blank in a conventional 1 cm-quartz cell was mounted into the center of the integrating sphere

and the excitation light was focused into the middle of the sample. The quantum yield was obtained from a Lecroy Wave Runner 6100 digital oscilloscope (1 GHz) using a tunable laser (pulse width = 4 ns, gate = 50 ns, Continuum Sunlite OPO) as excitation source under the excitation of 409 nm.

Two-photon approach to trigger CO release

Two-photon absorption is a way of accessing excited states by simultaneous absorption of two photons, in which the energy (or wavelength) of the photon is half the energy (or twice the wavelength) of the corresponding one-photon absorption. Fla displays high two-photon absorption near 800 nm and can be effectively excited by two-photon laser (Li et al., 2018). Due to the low phototoxicity and deep tissue penetration of two-photon technology, the two-photon irradiation was performed for photo-induced in situ CO release in the tissues. The paws of each rat were irradiated with two-photon laser for 15 min in a scanned manner (λ = 820 nm, using confocal laser source, 4100 mW/cm^2 at 4% laser power).

Cell culture

PC12 cells (rat pheochromocytoma cells, American Type Culture Collection, Manassas, VA, USA) were cultured in Iscove-modified Dulbecco's medium (IMDM, Gibco®, Life Technologies, Carlsbad, CA, USA) supplemented with 5% fetal bovine serum and 10% horse serum in a humidified 5% CO_2 environment at 37 °C.

MTT assays on PC12 cells

The cytotoxicity of the Fla and nanoparticles in the dark was evaluated using PC12 cells as the model cell line. Similar assays were performed for Neu-MnO_2/Fla under illumination conditions. For all experiments, MTT (Sigma-Aldrich) was prepared fresh at 5 mg/mL in sterile PBS. This solution was filtered through a 0.22 µm PES filter. PC12 cells were seeded in 96-well plates at 10,000 cells/well for 24 h. The cells were then treated with Fla at final concentrations of 0-50 uM with a final DMSO concentration that does not exceed 0.4% (v/v), and treated with nanoparticles at final

concentrations of 0-100 ug/mL, respectively. For illuminated group, the plates were illuminated for 1 h and incubated for an additional 23 h. After 24 h, MTT solution (20 µL) was added and the cells were incubated for an additional 4 h. The metabolized formazan pellets were solubilized by adding 100 µL of DMSO and absorption values were measured using Modulus™ Microplate reader (Turner Biosystems) at 570 nm and 630 nm. The cytotoxicity was expressed as the percentage of cell viability as compared with the blank control. The results are presented as means ± standard deviation from three independent experiments.

Neu-MnO_2/Fla adhesion assay

PC12 cells were seeded in 24-well plates and cultured overnight. Cell culture medium was changed and LPS was added to the desired concentrations. After 6 h of stimulation, cells were washed with PBS and blocked with 1% BSA for 1 h. The cells were then incubated with 25 ug/mL Neu-MnO_2/Fla and RBC-MnO_2/Fla in PBS at 4 °C for 10 min, respectively. After incubation, cells were washed five times with ice-cold PBS and fixed with 10% formalin for 10 min, then 4′, 6-diamidino-2-phenylindole (DAPI) solution (1 µg/mL) was added to stain the nuclei and the cells were visualized under a confocal laser scanning microscope.

Fluorescence microscopy of intracellular Fla

PC12 cells were seeded into 24-well plates at an initial density of 1.0 x 10^5 cells/cm^2 and allowed to adhere to the chamber slides for 24 hours. Neu-MnO_2/Fla dissolved in PBS buffer was added to a final concentration of 25 ug/mL per chamber. The cells were incubated for 6 hour in media, protected from light. Then, the cells were washed three times with PBS buffer and irradiated (λ = 410 nm; power = 15 mW/cm^2) for different periods of time, followed by treated with DAPI (1 µg/mL) for 15 min and three washes with PBS buffer to remove all the residual dye. The cells were visualized under a confocal laser scanning microscope. Acquired images were processed by universal adjustment to enhance contrast levels (same settings were applied for all acquired images in each detection channel).

Anti-inflammatory effect of Neu-MnO_2/Fla

PC12 cells were seeded in 24-well plates one day before the experiment. PC12 cells were pre-treated with Fla (25 uM, 0.1% (v/v) DMSO), Neu-MnO_2, or Neu-MnO_2/Fla (Fla = 25 uM) for 6 h. One of the two prepared plates was then subjected to illuminate (λ = 410 nm; power = 15 mW/cm^2) for 10 min at 37 °C. The second plate remained in the incubator in the dark for the same amount of time. Thereafter, LPS (1 μg/mL final concentration) was added into the cell culture media to stimulate the inflammatory response of PC12 cells. The plates were then incubated at 37 °C for 1 h. The supernatants were collected and centrifugated for 5 min at 10,000 rpm to remove particles. The levels of TNF-α and IL-1β were measured using corresponding ELISA kits. Data were analyzed to compare effects of all treatments to the LPS control or to compare effects of all treatment under illuminated and non-illuminated conditions. Quantitative data for the expression of TNF-α and IL-1β levels in all groups was performed. The results are presented as means ± standard deviation from three independent experiments.

Animal study

Wild-type Wistar rats were chosen as test animals, in a weight range of 200-250 g (8-12 weeks old) and random in sex. All animal studies were conducted in accordance with the principles and procedures outlined in "Regulations for the Administration of Affairs Concerning Laboratory Animals", approved by the National Council of China on October 31, 1988, and "The National Regulation of China for Care and Use of Laboratory Animals", promulgated by the National Science and Technology Commission of China, on November 14, 1988 as Decree No. 2. Protocols were approved by the Committee of Jilin University Institutional Animal Care and Use.

To induce tissue inflammation, the rear paws of each rat were stimulated using LPS (200 μL, 1 mg/mL) via local injection. At 6 h following LPS stimulation, the rats were anesthetized with 10% chloral hydrate and subcutaneously injected with Neu-MnO_2/Fla to the inflamed paws (at a dose of 25 ug/mL). Fla displays high

two-photon absorption near 800 nm and can be effectively excited by two-photon laser (Li et al., 2018). Due to the low phototoxicity and deep tissue penetration of two-photon technology, after 4 h of incubation, two-photon irradiation was performed for photo-induced in situ CO release in the tissues. The paws of each rat were irradiated with two-photon laser for 15 min in a scanned manner (λ = 820 nm, using confocal laser source, 4100 mW/cm^2 at 4% laser power). After 1 h of incubation, changes in the levels of ROS in the inflamed tissues were evaluated using a luminescent probe, DCFH-DA (1 mM, 100 uL), which was administered by subcutaneous injection in a similar way. Then, luminescence images of the inflamed paws were then acquired using an IVIS imaging system.

After the animals were sacrificed, the inflamed paws were harvested. Tissue specimens from the harvested paws were homogenized in 10% NP40 and centrifuged at 14,000 rpm for 10 min at 4 °C. The obtained supernatants were then used to analyze the levels of TNF-α and IL-1β that were expressed in the inflamed tissues, using the aforementioned ELISA kits. Additional tissue specimens were fixed in 10% formalin for 24 h and stained with H&E for histological examination. All results are presented as mean ± standard deviation.

SUPPLEMENTAL REFERENCES

ISSEKUTZ, A. C. & ISSEKUTZ, T. B. (1993). A major portion of polymorphonuclear leukocyte and T lymphocyte migration to arthritic joints in the rat is via LFA-1/MAC-1-independent mechanisms. *Clin. Immunol. Immunopathol.* 67**,** 257-63.

KUMAR, S., JYOTI, A., KESHARI, R. S., SINGH, M., BARTHWAL, M. K. & DIKSHIT, M. (2010). Functional and molecular characterization of NOS isoforms in rat neutrophil precursor cells. *Cytom. Part A.* 77**,** 467-77.

LEE, M. Y., KUAN, Y. H., CHEN, H. Y., CHEN, T. Y., CHEN, S. T., HUANG, C. C., YANG, I. P., HSU, Y. S., WU, T. S. & LEE, E. J. (2007). Intravenous administration of melatonin reduces the intracerebral cellular inflammatory response following transient focal cerebral ischemia in rats. *J. Pineal Res.* 42**,** 297-309.

LI, Y., SHU, Y., LIANG, M., XIE, X., JIAO, X., WANG, X. & TANG, B. (2018). A two-photon H_2O_2-activated CO photoreleaser. *Angew. Chem. Int. Ed.* 57**,** 12415-12419.

MERT, T., SAHIN, M., SAHIN, E. & YAMAN, S. (2019). Anti-inflammatory properties of liposome-encapsulated clodronate or Anti-Ly6G can be modulated by peripheral or central inflammatory markers in carrageenan-induced inflammation model. *Inflammopharmacology* 27**,** 603-612.

PAVLOVIC, M. D., COLIC, M., PEJNOVIC, N., TAMATANI, T., MIYASAKA, M. & DUJIC, A. (1994). A novel anti-rat CD18 monoclonal antibody triggers lymphocyte homotypic aggregation and granulocyte adhesion to plastic: different intracellular signaling pathways in resting versus activated thymocytes. *Eur. J. Immunol.* 24**,** 1640-8.

WANG, Y., HAN, B., CHEN, N., DENG, D., GUAN, H. & WANG, Y. (2016). Enhanced microwave absorption properties of MnO_2 hollow microspheres consisted of MnO_2 nanoribbons synthesized by a facile hydrothermal method. *J. Alloys Compd.* 676**,** 224-230.

ZHANG, L., WANG, Z., ZHANG, Y., CAO, F., DONG, K., REN, J. & QU, X. (2018). Erythrocyte membrane cloaked metal-organic framework nanoparticle as biomimetic nanoreactor for starvation-activated colon cancer therapy. *ACS. Nano.* 12**,** 10201-10211.

ZHANG, Q., DEHAINI, D., ZHANG, Y., ZHOU, J., CHEN, X., ZHANG, L., FANG, R. H., GAO, W. & ZHANG, L. (2018). Neutrophil membrane-coated nanoparticles inhibit synovial inflammation and alleviate joint damage in inflammatory arthritis. *Nat. nanotechnol.* 13**,** 1182-1190.
